# Supplementary material for: Interferon-γ selectively promotes survival of alveolar progenitor cells in a human lung organoid model
Source: EMBO J. 2026 Apr 16;45(10):3364–95. doi: 10.1038/s44318-026-00774-4 (PMC13187038; doi:10.1038/s44318-026-00774-4)
Supplement: Supplementary file 7 — Source data Fig. 1 [file 44318_2026_774_MOESM7_ESM.zip › Figure 1/F/Flow analysis with percentages.pdf]

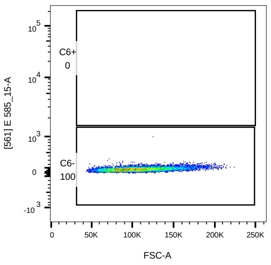

250731 sort AT2 and AT0 CEACAM6_Specimen_001_NEG_001.fcs
DAPI-
9654

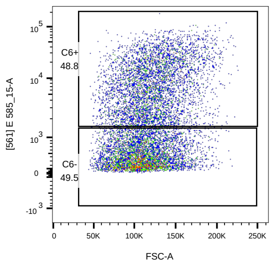

250731 sort AT2 and AT0 CEACAM6_Specimen_001_37_005.fcs
DAPI-
9510

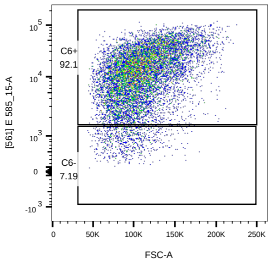

250731 sort AT2 and AT0 CEACAM6_Specimen_001_38_006.fcs
DAPI-
9540

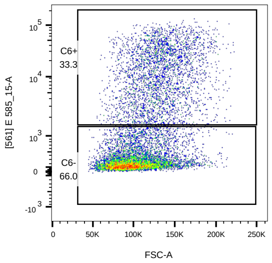

250731 sort AT2 and AT0 CEACAM6_Specimen_001_53_003.fcs
DAPI-
9385

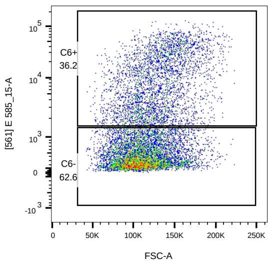

250731 sort AT2 and AT0 CEACAM6_Specimen_001_54_004.fcs
DAPI-
9508

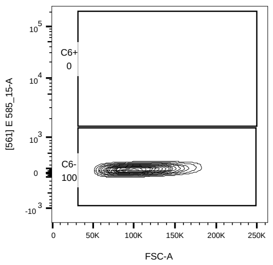

250731 sort AT2 and AT0 CEACAM6_Specimen_001_NEG_001.fcs
DAPI-
9654

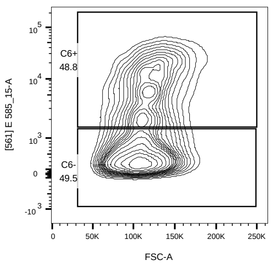

250731 sort AT2 and AT0 CEACAM6_Specimen_001_37_005.fcs
DAPI-
9510

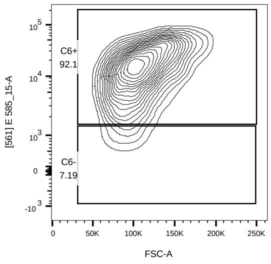

250731 sort AT2 and AT0 CEACAM6_Specimen_001_38_006.fcs
DAPI-
9540

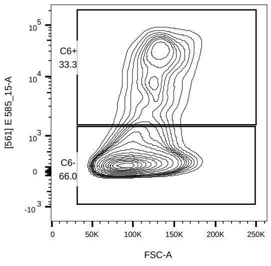

250731 sort AT2 and AT0 CEACAM6_Specimen_001_53_003.fcs
DAPI-
9385

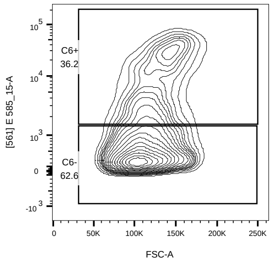

250731 sort AT2 and AT0 CEACAM6_Specimen_001_54_004.fcs
DAPI-
9508
