# Supplementary figures and images for: Interferon-γ selectively promotes survival of alveolar progenitor cells in a human lung organoid model
Source: EMBO J. 2026 Apr 16;45(10):3364–95. doi: 10.1038/s44318-026-00774-4 (PMC13187038; doi:10.1038/s44318-026-00774-4)

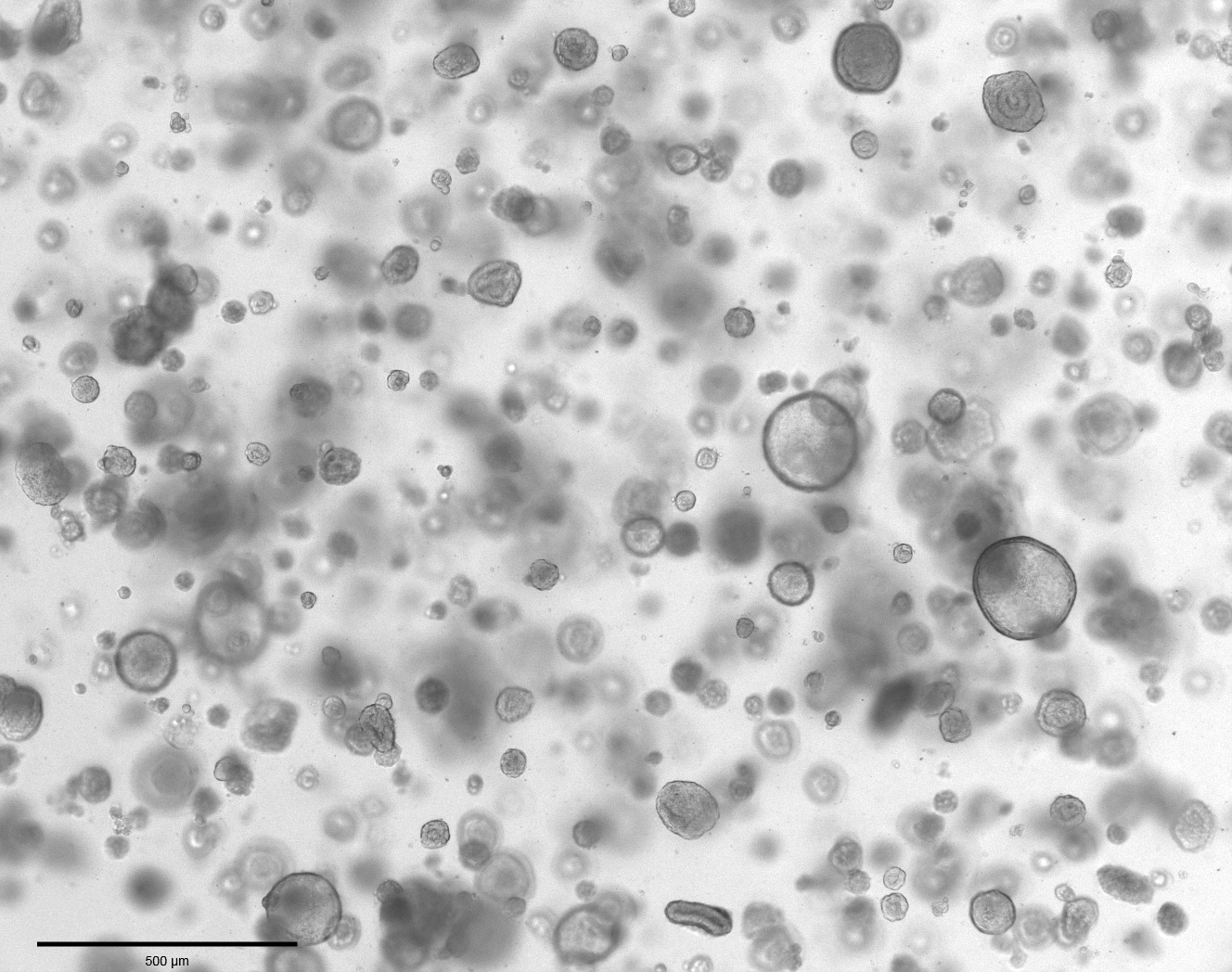

Supplement: Supplementary file 7 — Source data Fig. 1 [file 44318_2026_774_MOESM7_ESM.zip › Figure 1/I/4x 54 C6+.TIF]

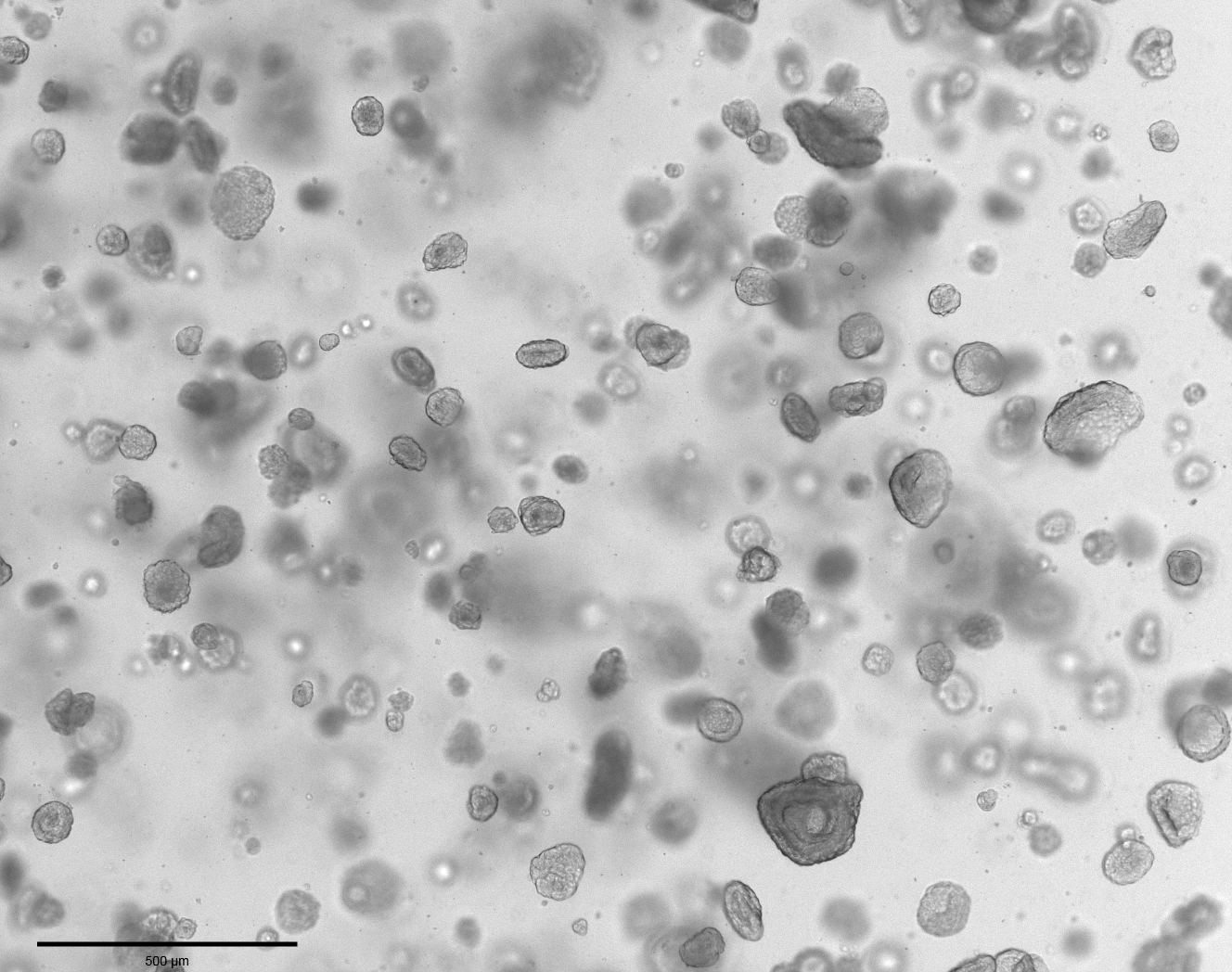

Supplement: Supplementary file 7 — Source data Fig. 1 [file 44318_2026_774_MOESM7_ESM.zip › Figure 1/I/4x 53 C6-.TIF]

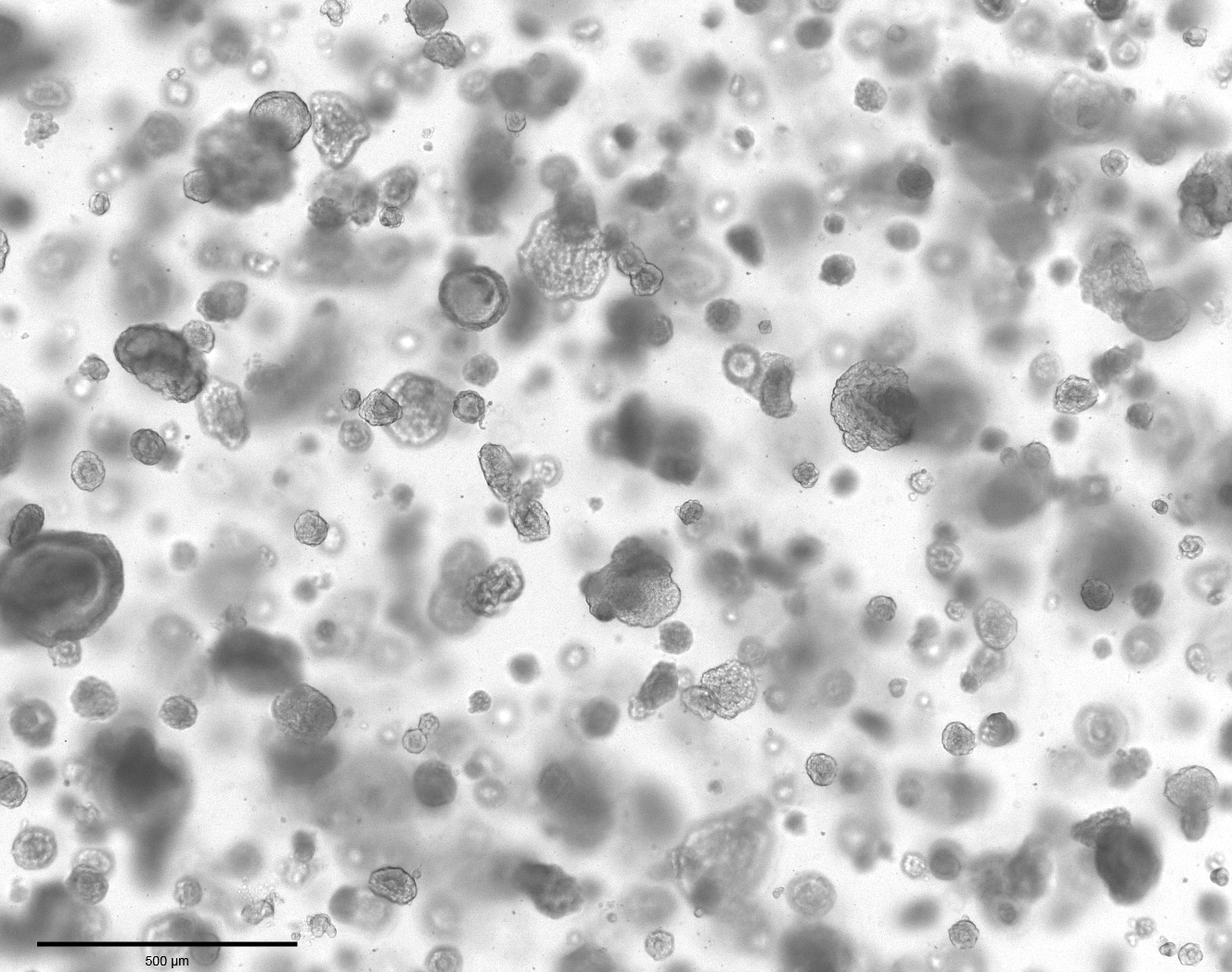

Supplement: Supplementary file 7 — Source data Fig. 1 [file 44318_2026_774_MOESM7_ESM.zip › Figure 1/I/4x 54 C6-TIF.TIF]

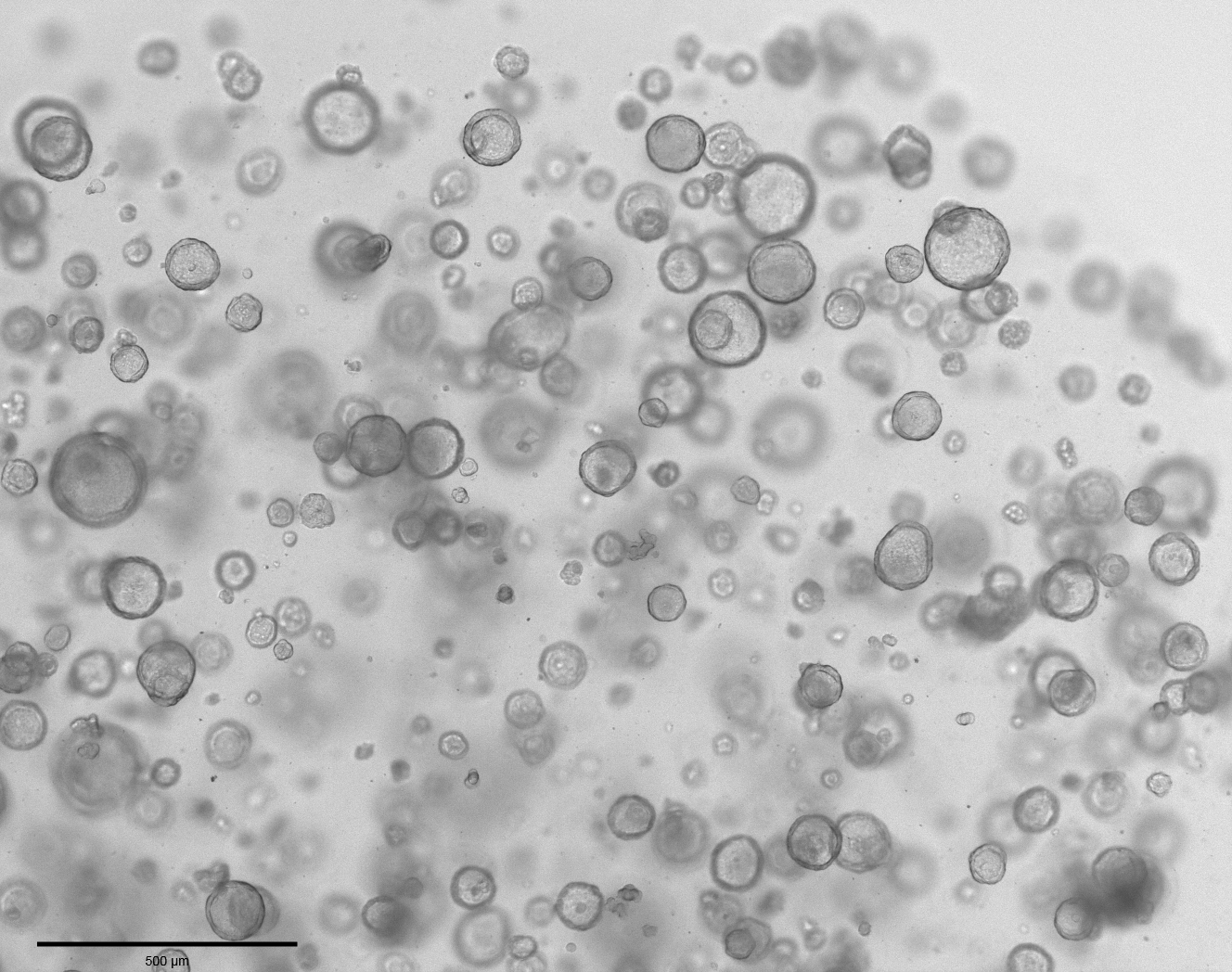

Supplement: Supplementary file 7 — Source data Fig. 1 [file 44318_2026_774_MOESM7_ESM.zip › Figure 1/I/4x 53 C6+.TIF]

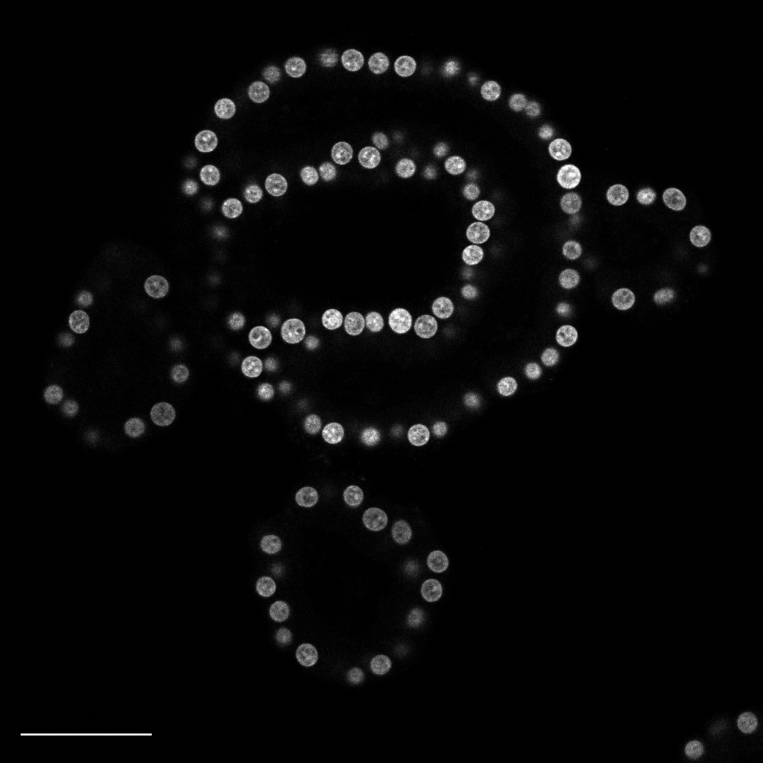

Supplement: Supplementary file 7 — Source data Fig. 1 [file 44318_2026_774_MOESM7_ESM.zip › Figure 1/E/DAPI.tif]

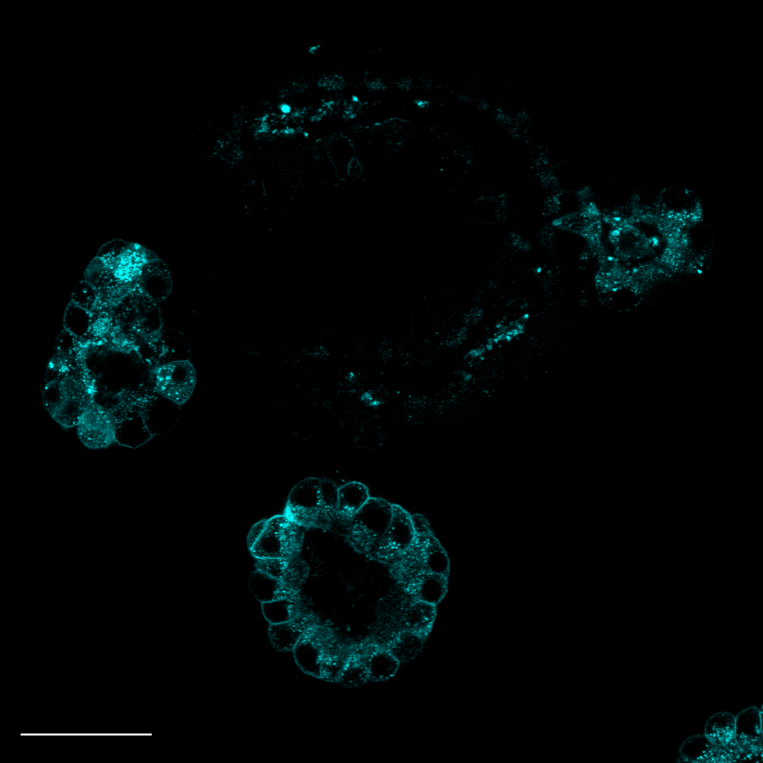

Supplement: Supplementary file 7 — Source data Fig. 1 [file 44318_2026_774_MOESM7_ESM.zip › Figure 1/E/CEACAM6.tif]

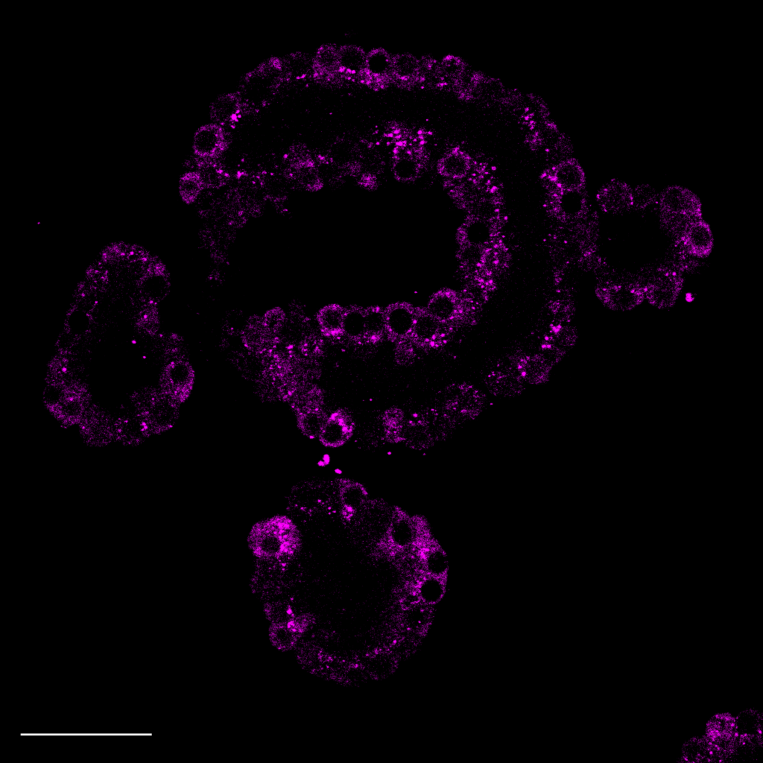

Supplement: Supplementary file 7 — Source data Fig. 1 [file 44318_2026_774_MOESM7_ESM.zip › Figure 1/E/SFTPC.tif]

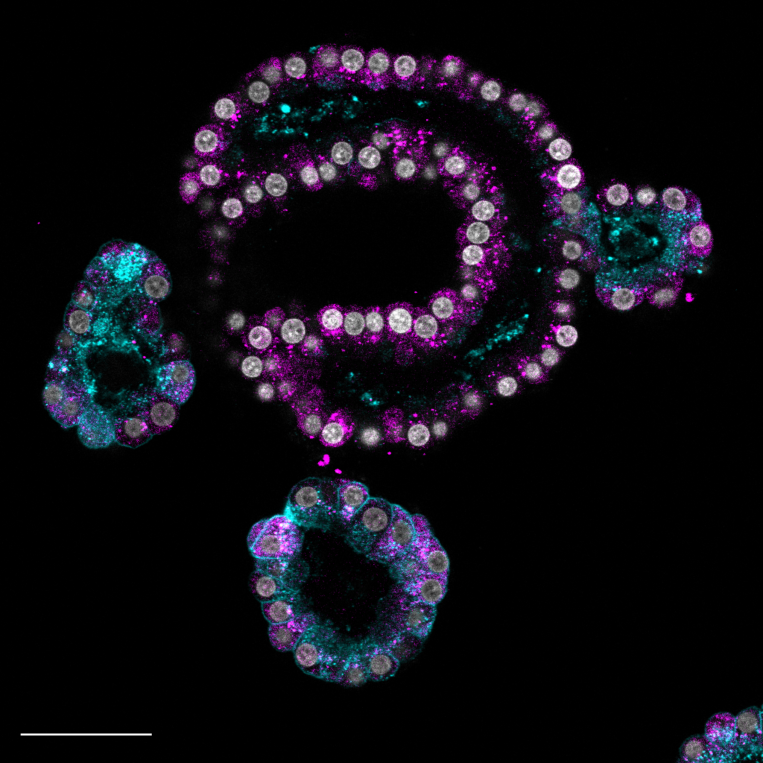

Supplement: Supplementary file 7 — Source data Fig. 1 [file 44318_2026_774_MOESM7_ESM.zip › Figure 1/E/merge.tif]

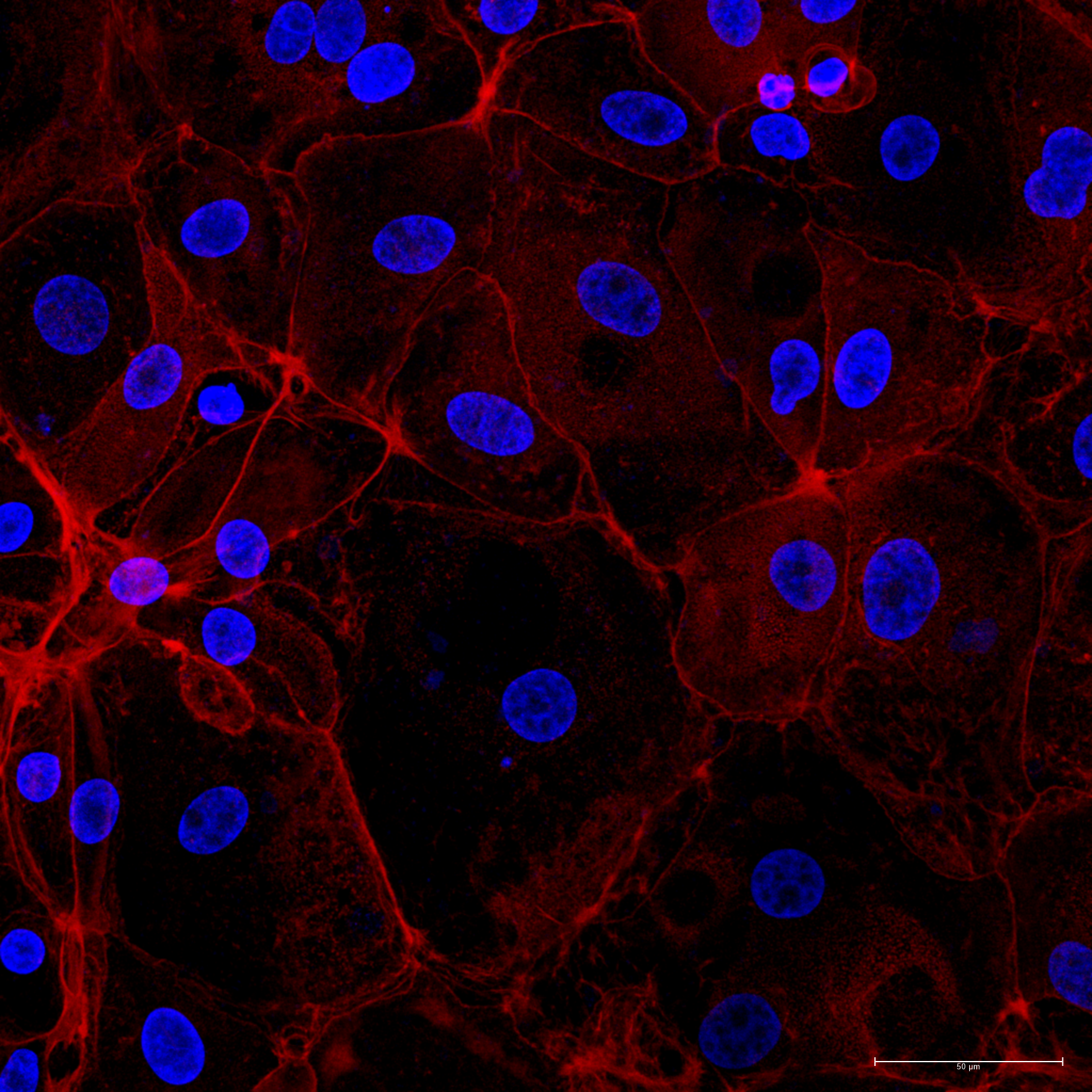

Supplement: Supplementary file 9 — Source data Fig. 3 [file 44318_2026_774_MOESM9_ESM.zip › Figure 3/G/invasin.tif]

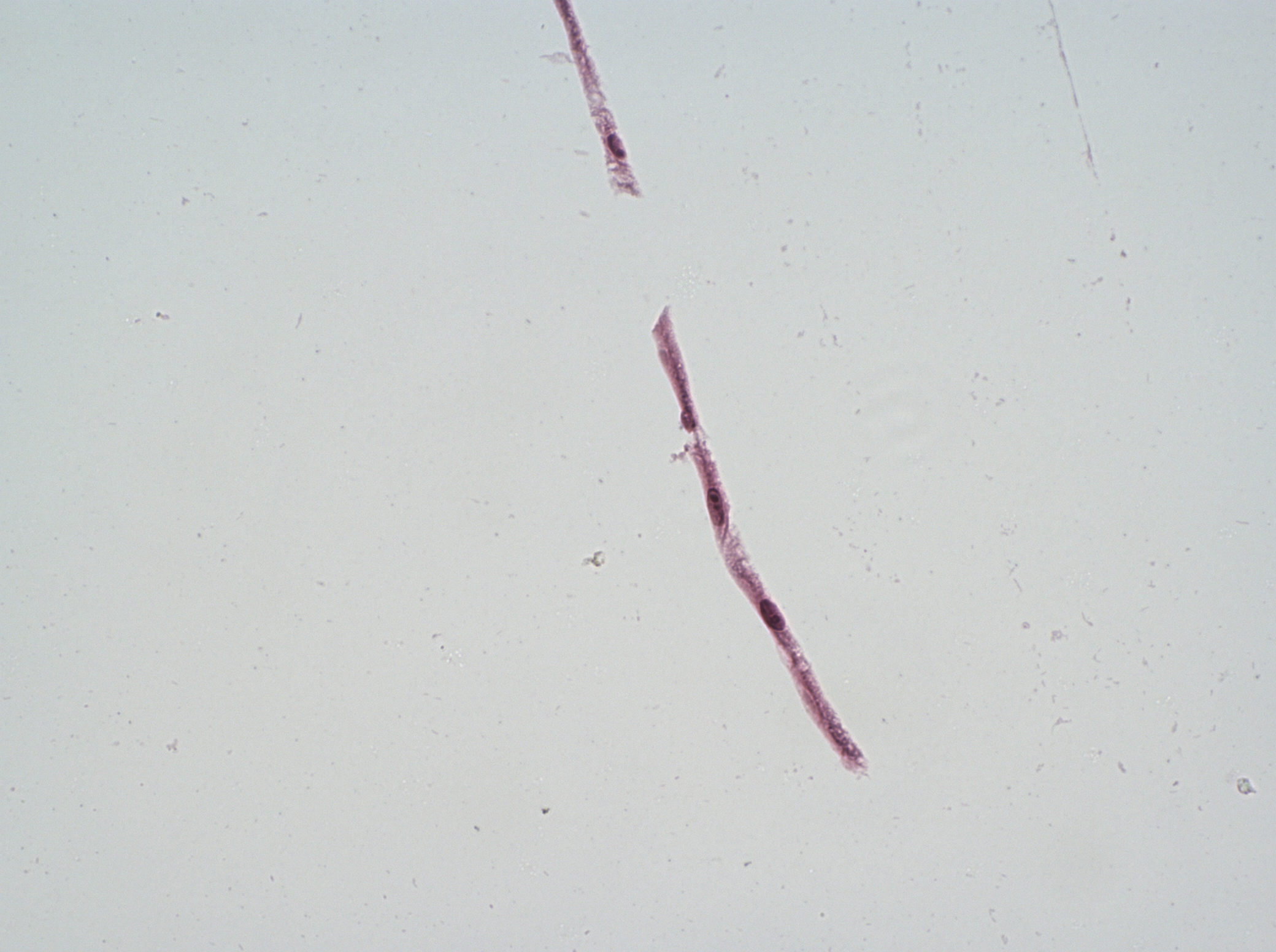

Supplement: Supplementary file 9 — Source data Fig. 3 [file 44318_2026_774_MOESM9_ESM.zip › Figure 3/G/HE invasin.TIF]

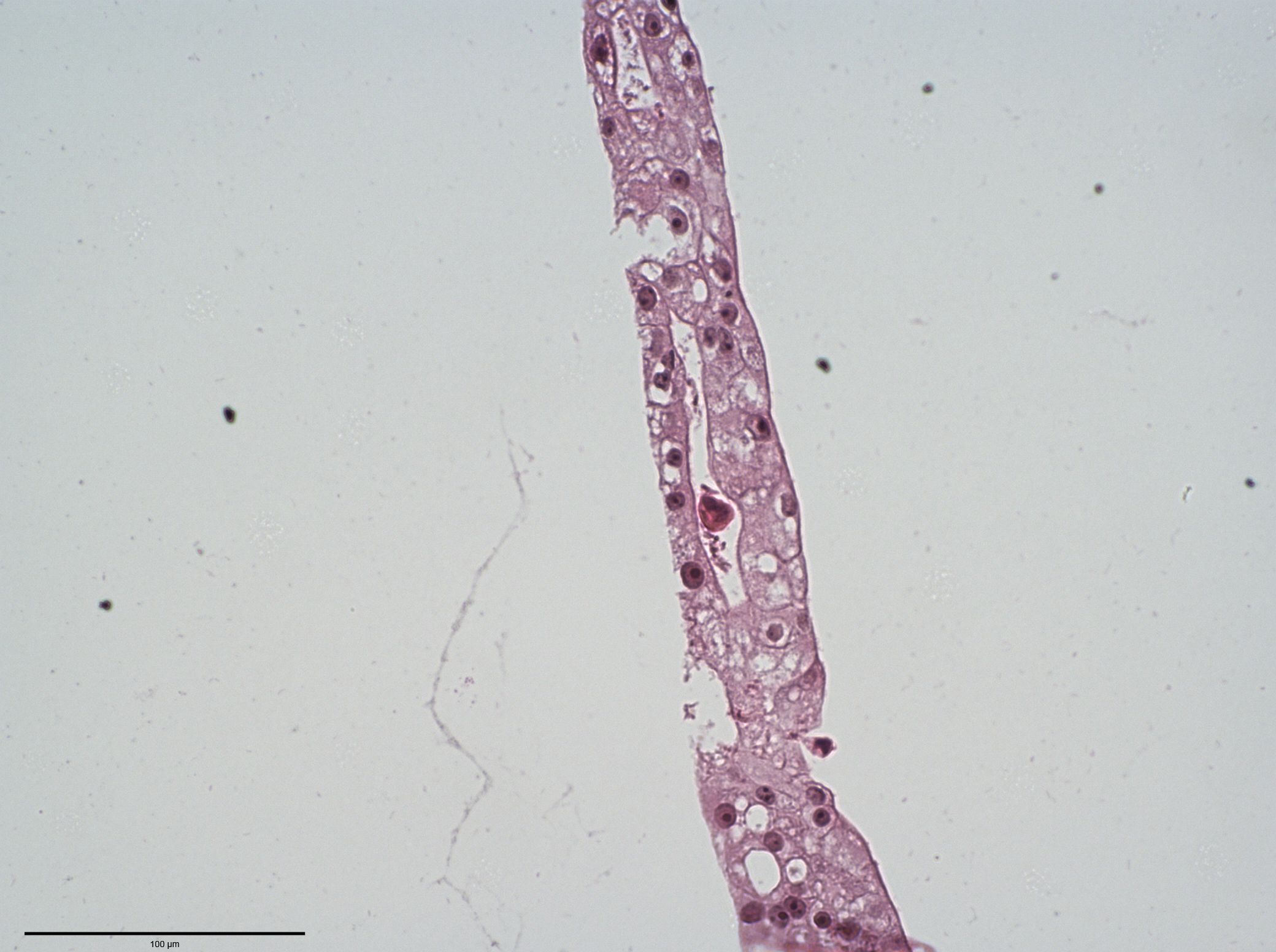

Supplement: Supplementary file 9 — Source data Fig. 3 [file 44318_2026_774_MOESM9_ESM.zip › Figure 3/G/HE BME.TIF]

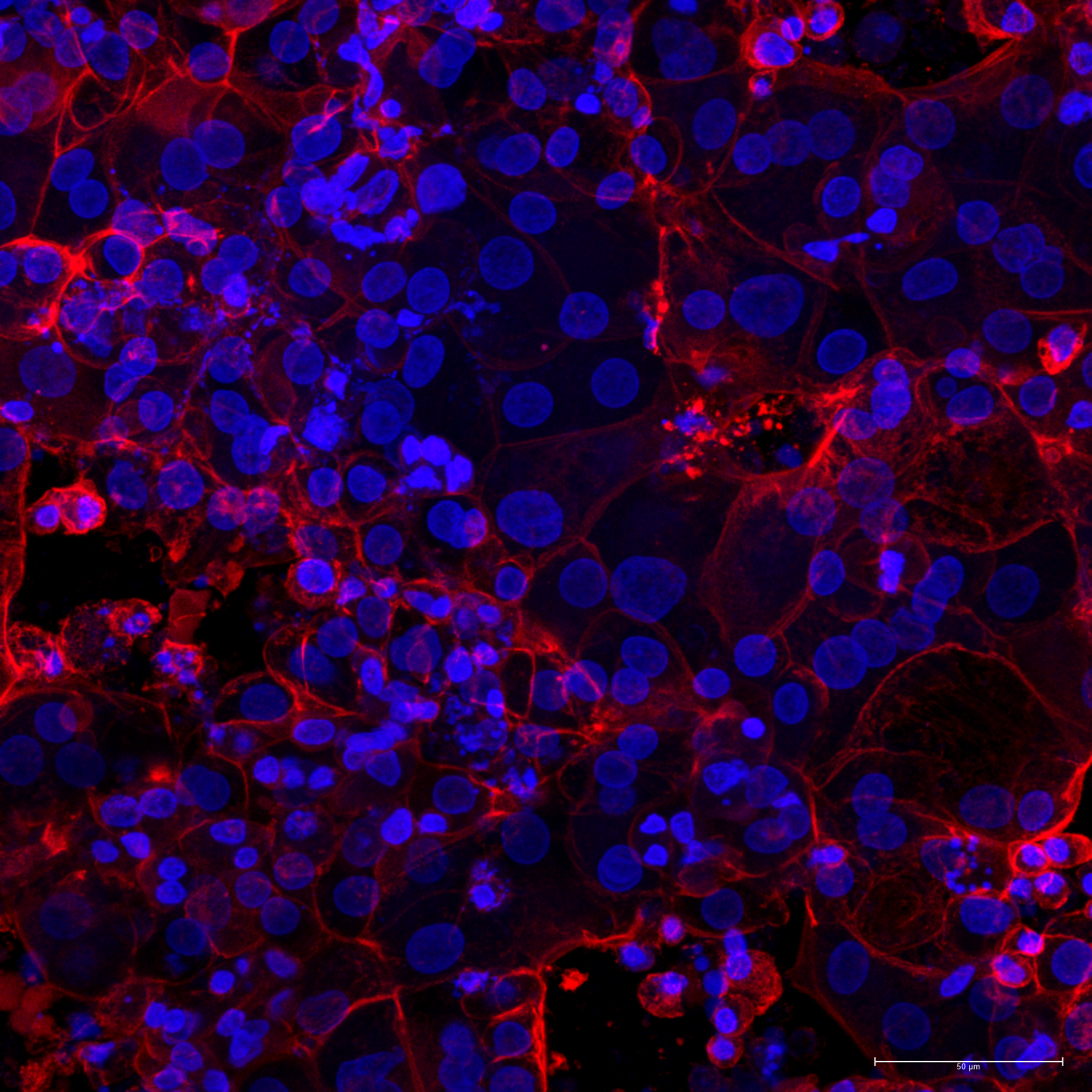

Supplement: Supplementary file 9 — Source data Fig. 3 [file 44318_2026_774_MOESM9_ESM.zip › Figure 3/G/BME.tif]

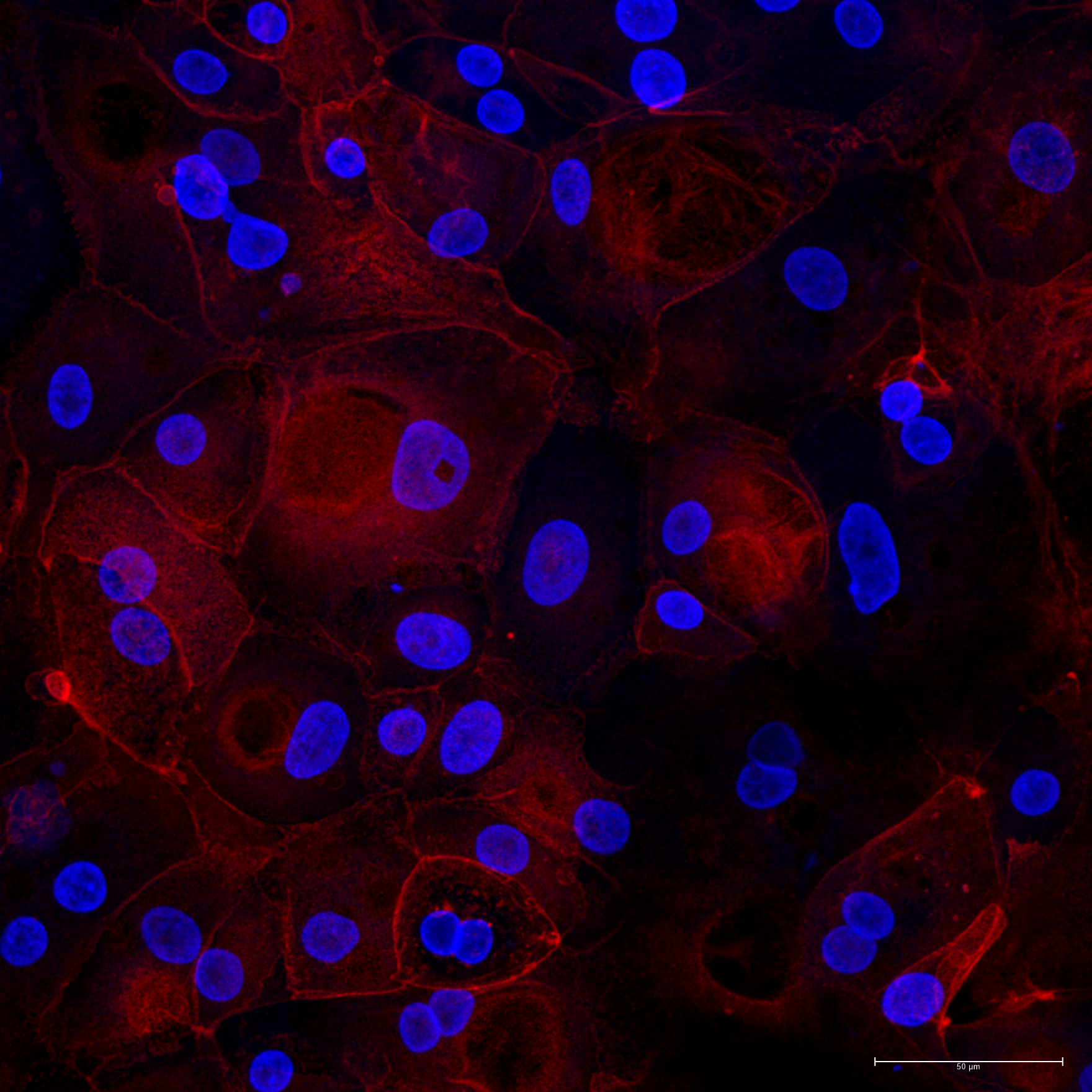

Supplement: Supplementary file 9 — Source data Fig. 3 [file 44318_2026_774_MOESM9_ESM.zip › Figure 3/G/collagen.tif]

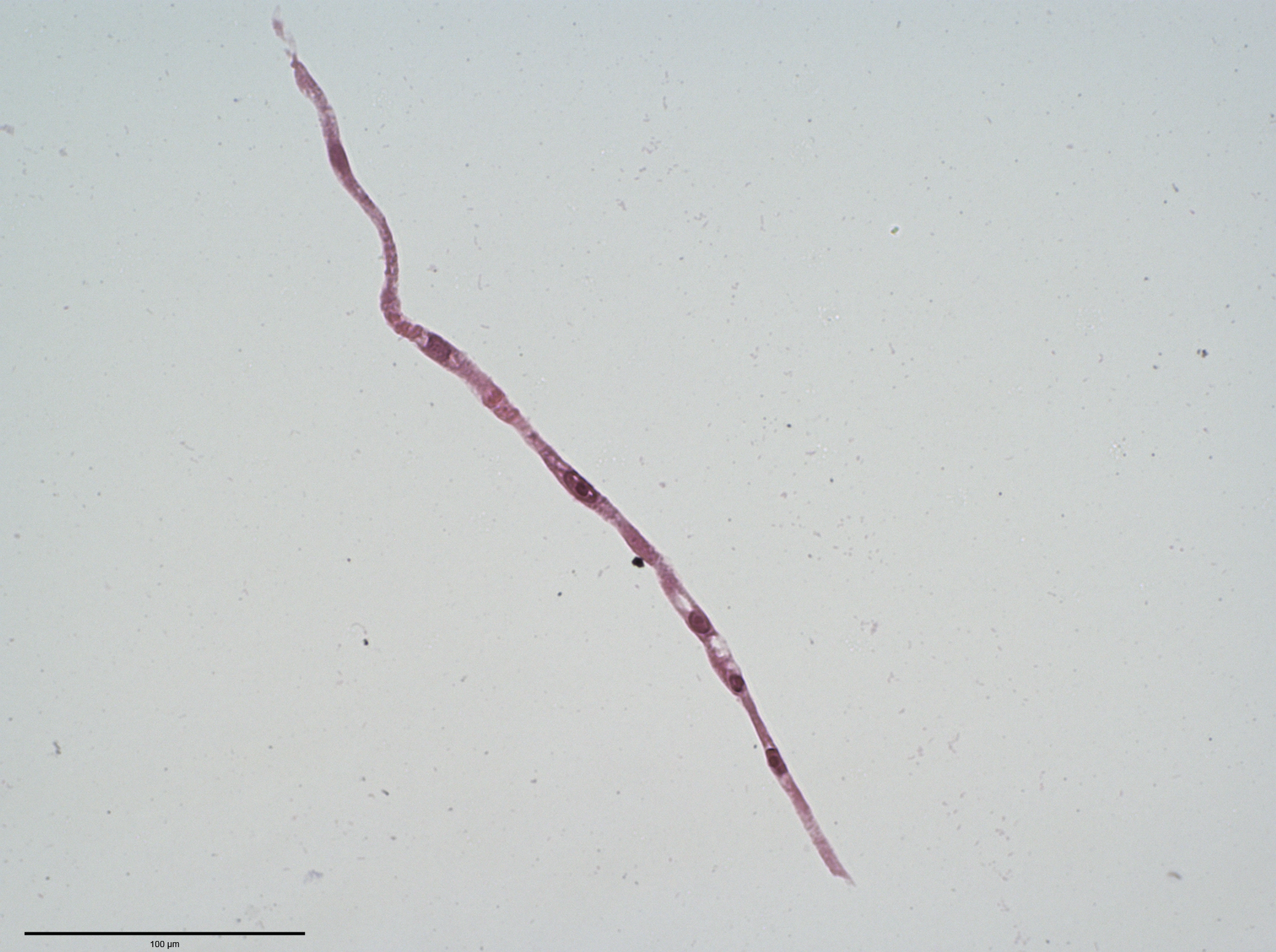

Supplement: Supplementary file 9 — Source data Fig. 3 [file 44318_2026_774_MOESM9_ESM.zip › Figure 3/G/HE collagen.TIF]

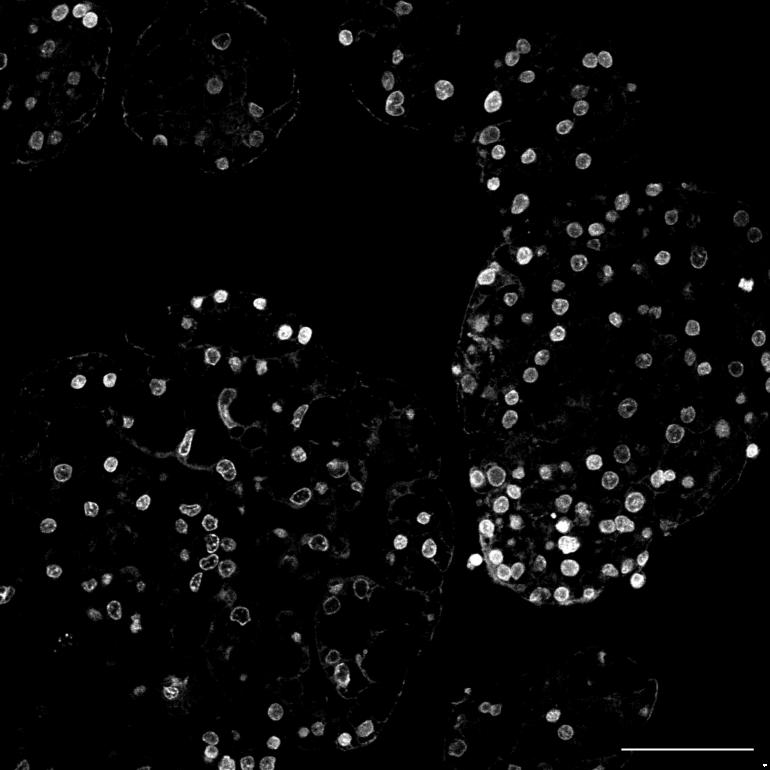

Supplement: Supplementary file 9 — Source data Fig. 3 [file 44318_2026_774_MOESM9_ESM.zip › Figure 3/F/DAPI.tif]

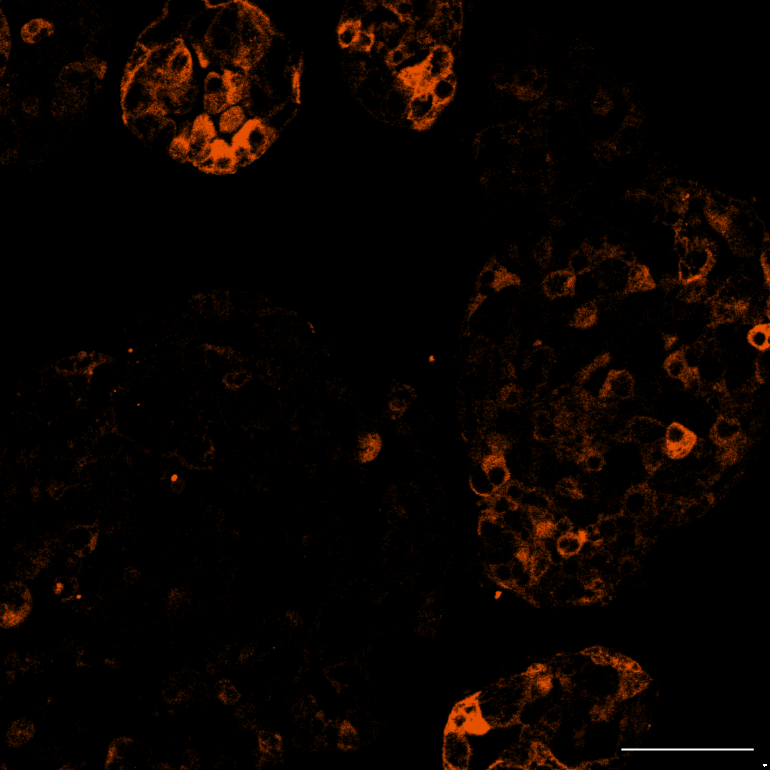

Supplement: Supplementary file 9 — Source data Fig. 3 [file 44318_2026_774_MOESM9_ESM.zip › Figure 3/F/AGER.tif]

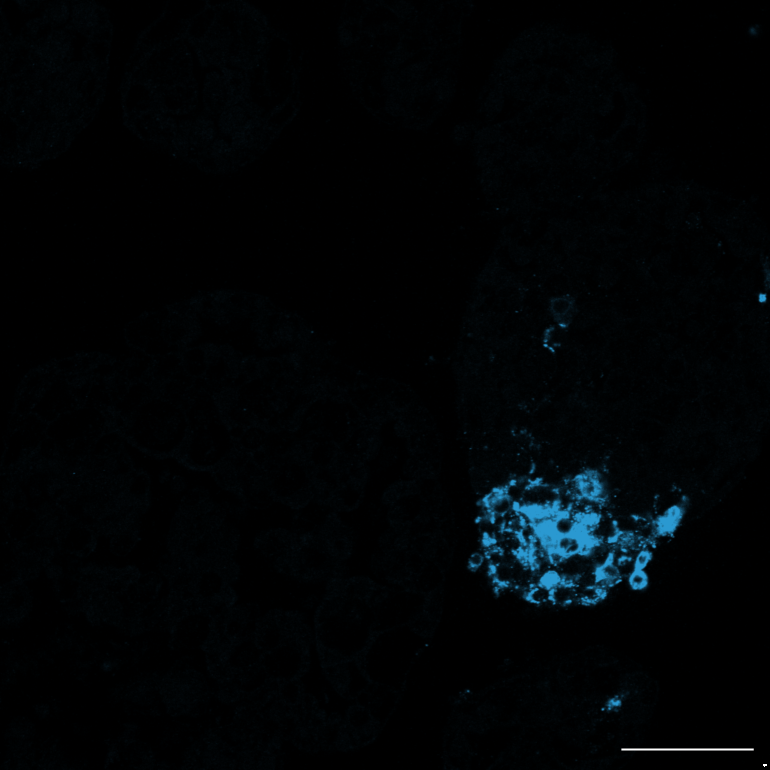

Supplement: Supplementary file 9 — Source data Fig. 3 [file 44318_2026_774_MOESM9_ESM.zip › Figure 3/F/SFTPC.tif]

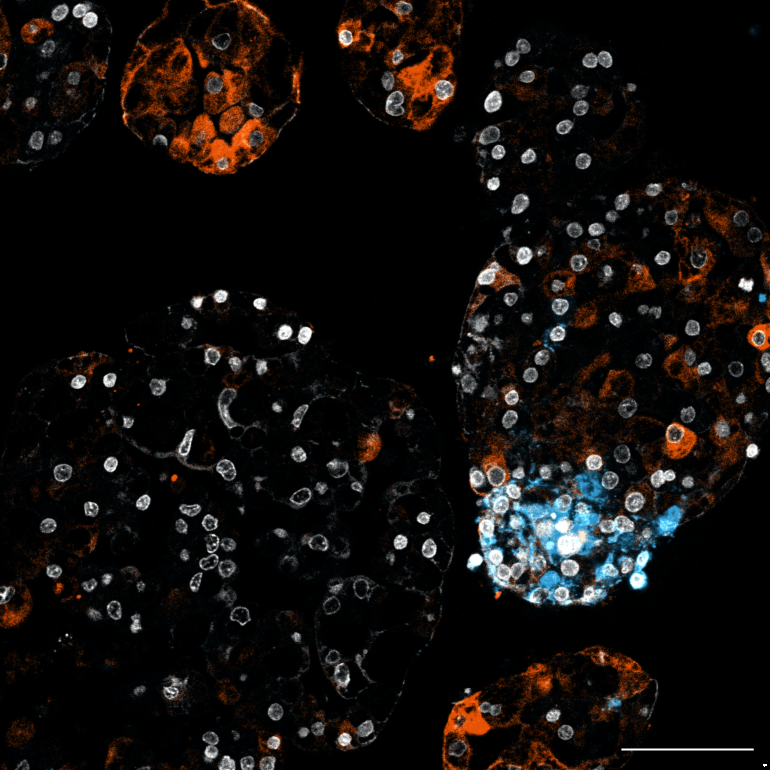

Supplement: Supplementary file 9 — Source data Fig. 3 [file 44318_2026_774_MOESM9_ESM.zip › Figure 3/F/merge.tif]

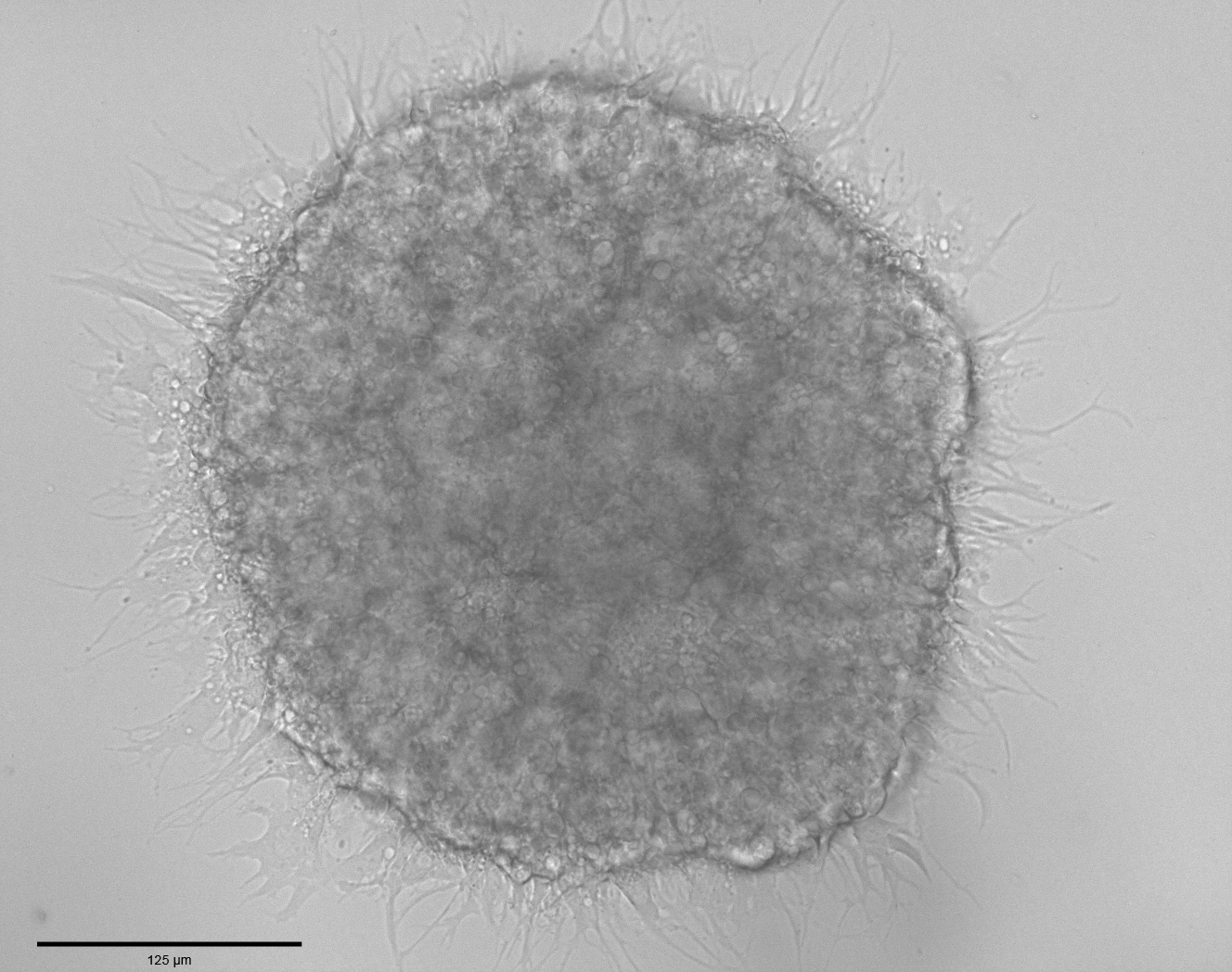

Supplement: Supplementary file 9 — Source data Fig. 3 [file 44318_2026_774_MOESM9_ESM.zip › Figure 3/C/Organoid.TIF]

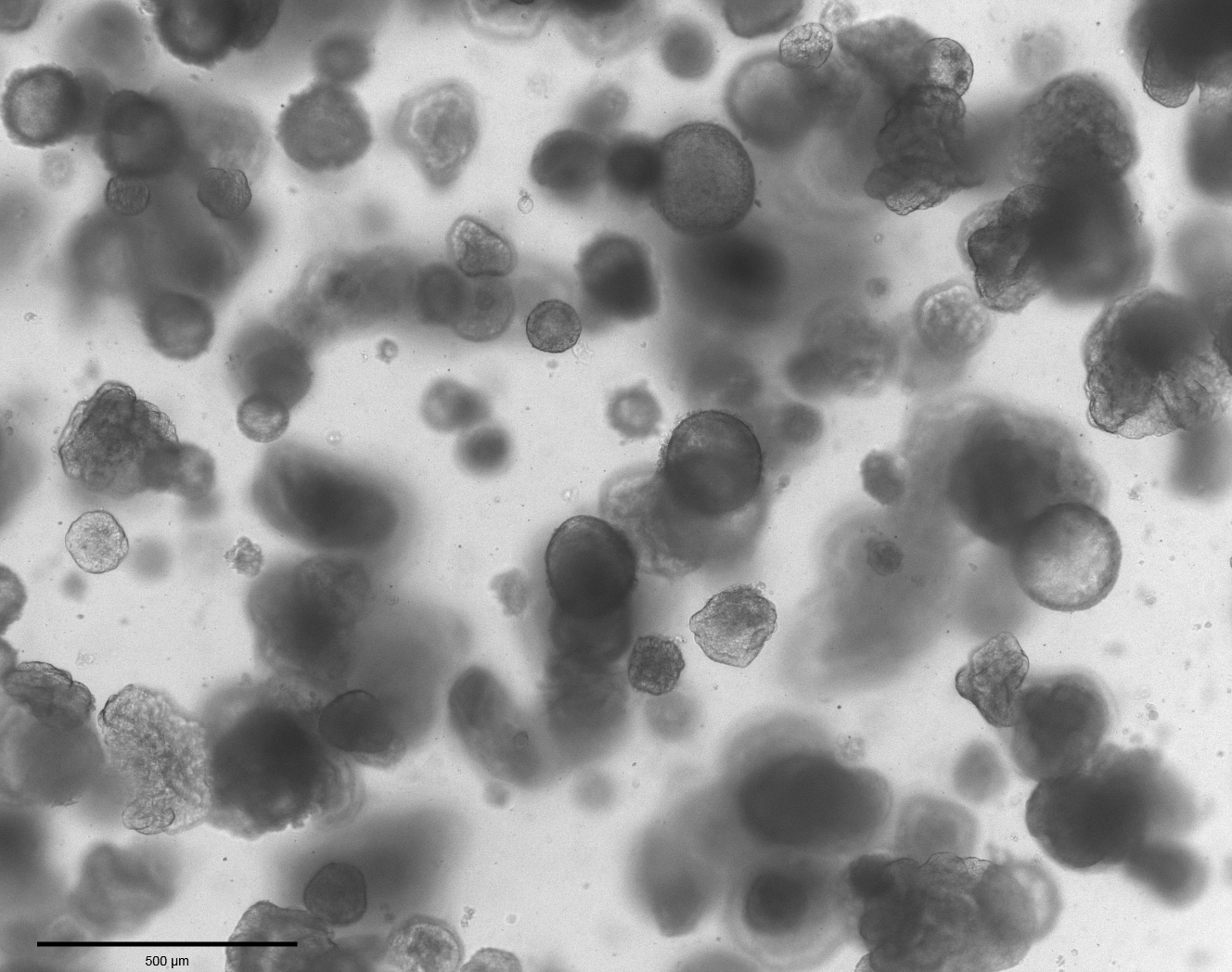

Supplement: Supplementary file 9 — Source data Fig. 3 [file 44318_2026_774_MOESM9_ESM.zip › Figure 3/B/AT1-2-M.TIF]

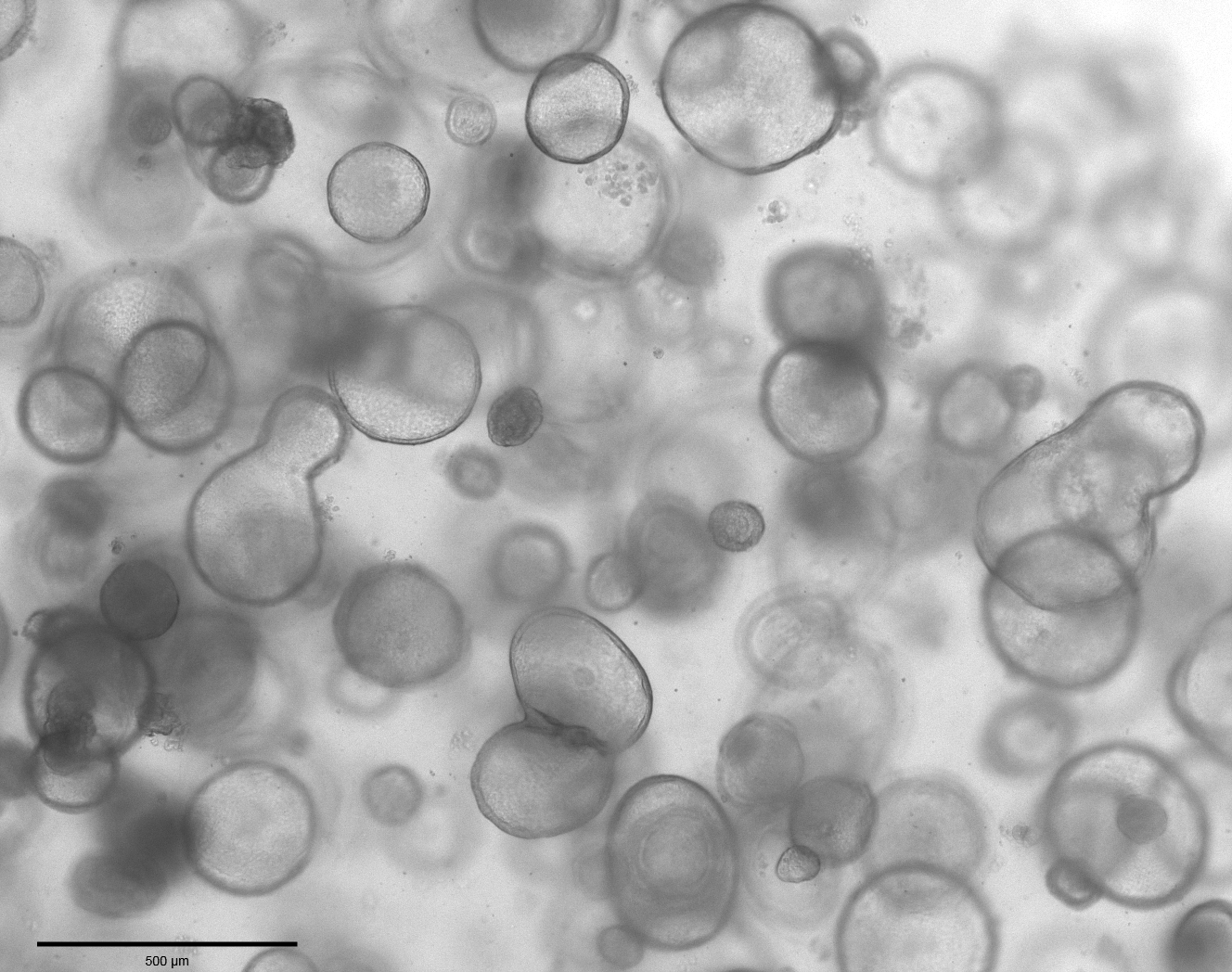

Supplement: Supplementary file 9 — Source data Fig. 3 [file 44318_2026_774_MOESM9_ESM.zip › Figure 3/B/ALVO-EM.TIF]

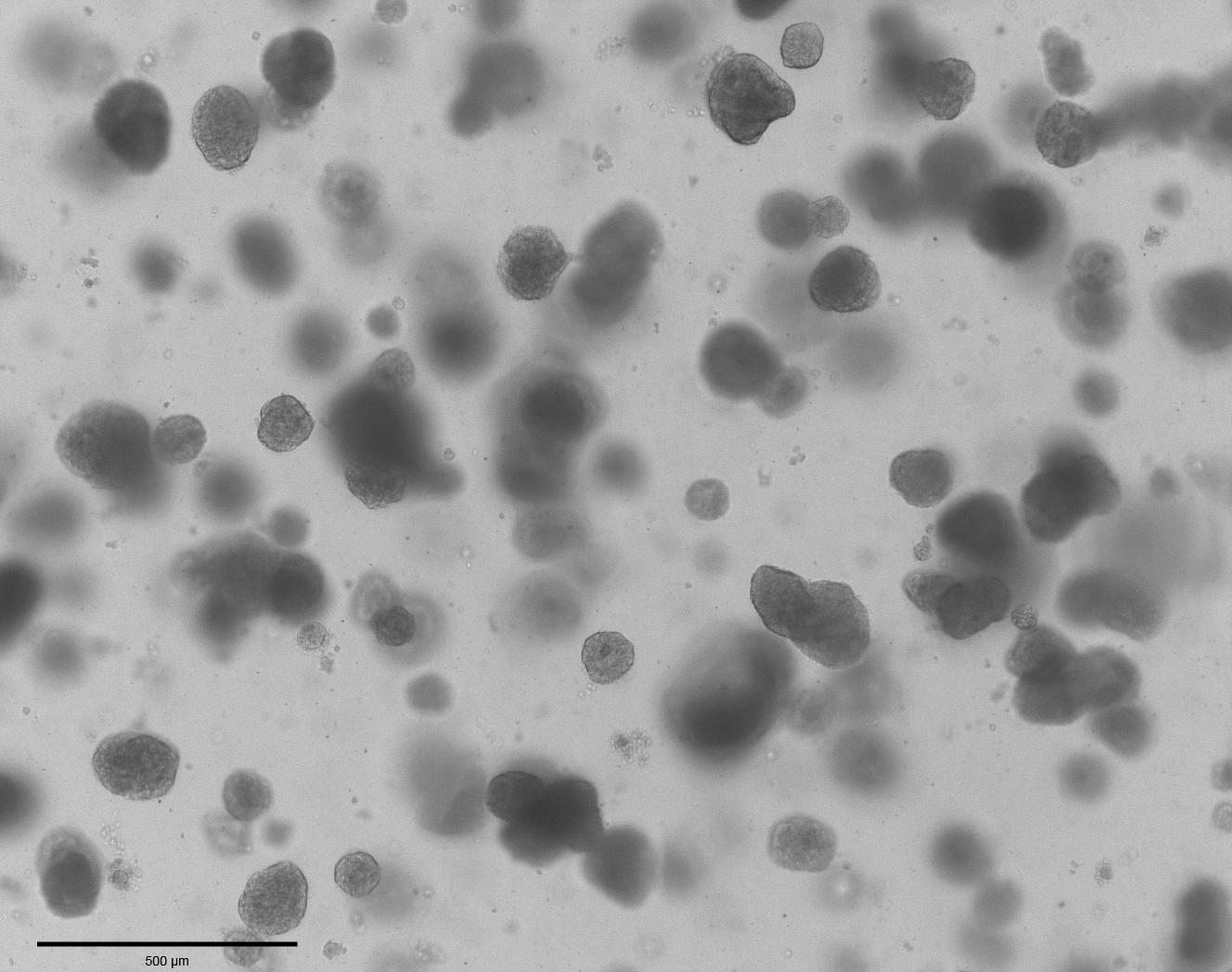

Supplement: Supplementary file 9 — Source data Fig. 3 [file 44318_2026_774_MOESM9_ESM.zip › Figure 3/B/BM+LATSi.TIF]

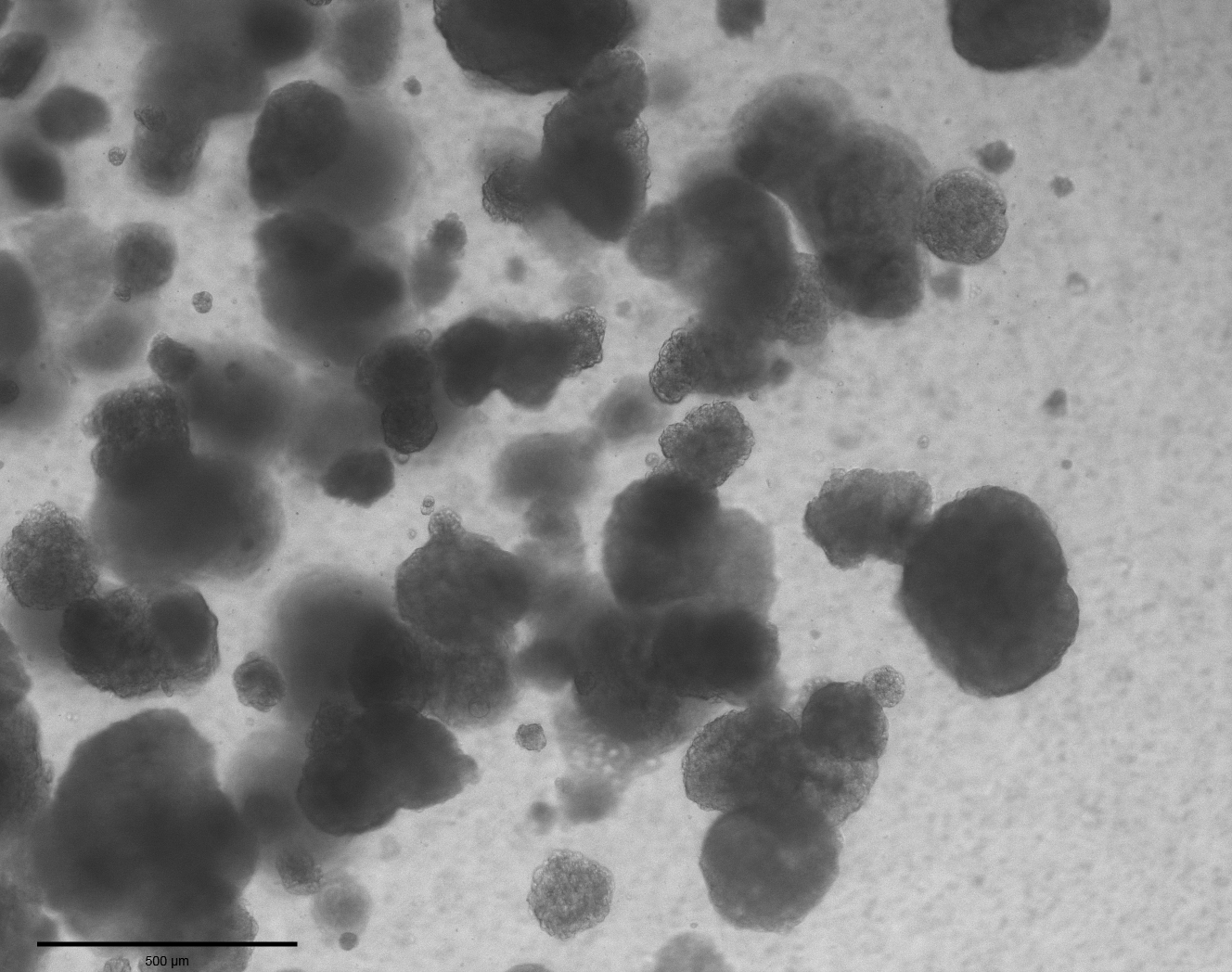

Supplement: Supplementary file 11 — Source data Fig. 6 [file 44318_2026_774_MOESM11_ESM.zip › Figure 6/C/untreated MP AT1:2-M.TIF]

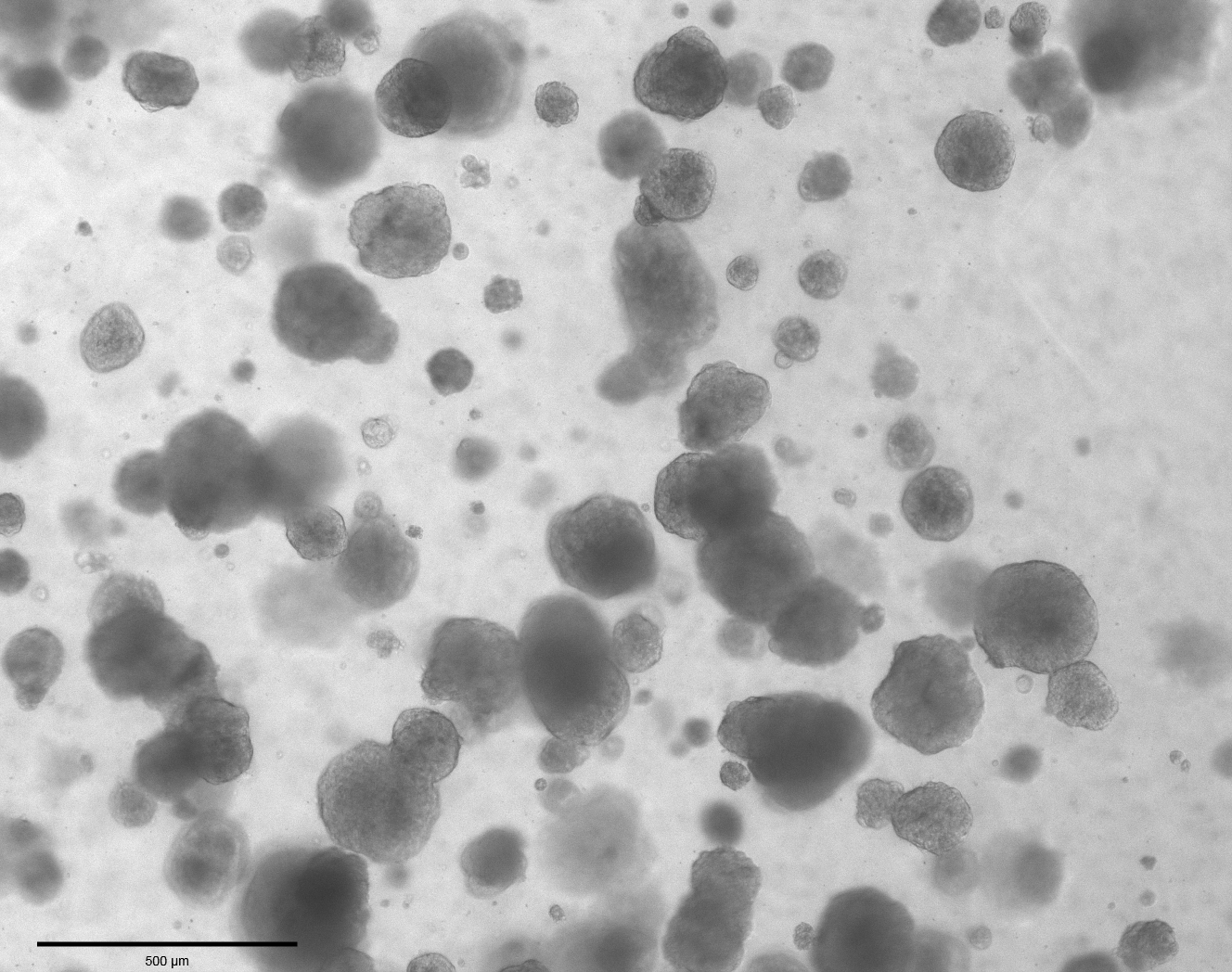

Supplement: Supplementary file 11 — Source data Fig. 6 [file 44318_2026_774_MOESM11_ESM.zip › Figure 6/C/treated MP AT1:2-M.TIF]

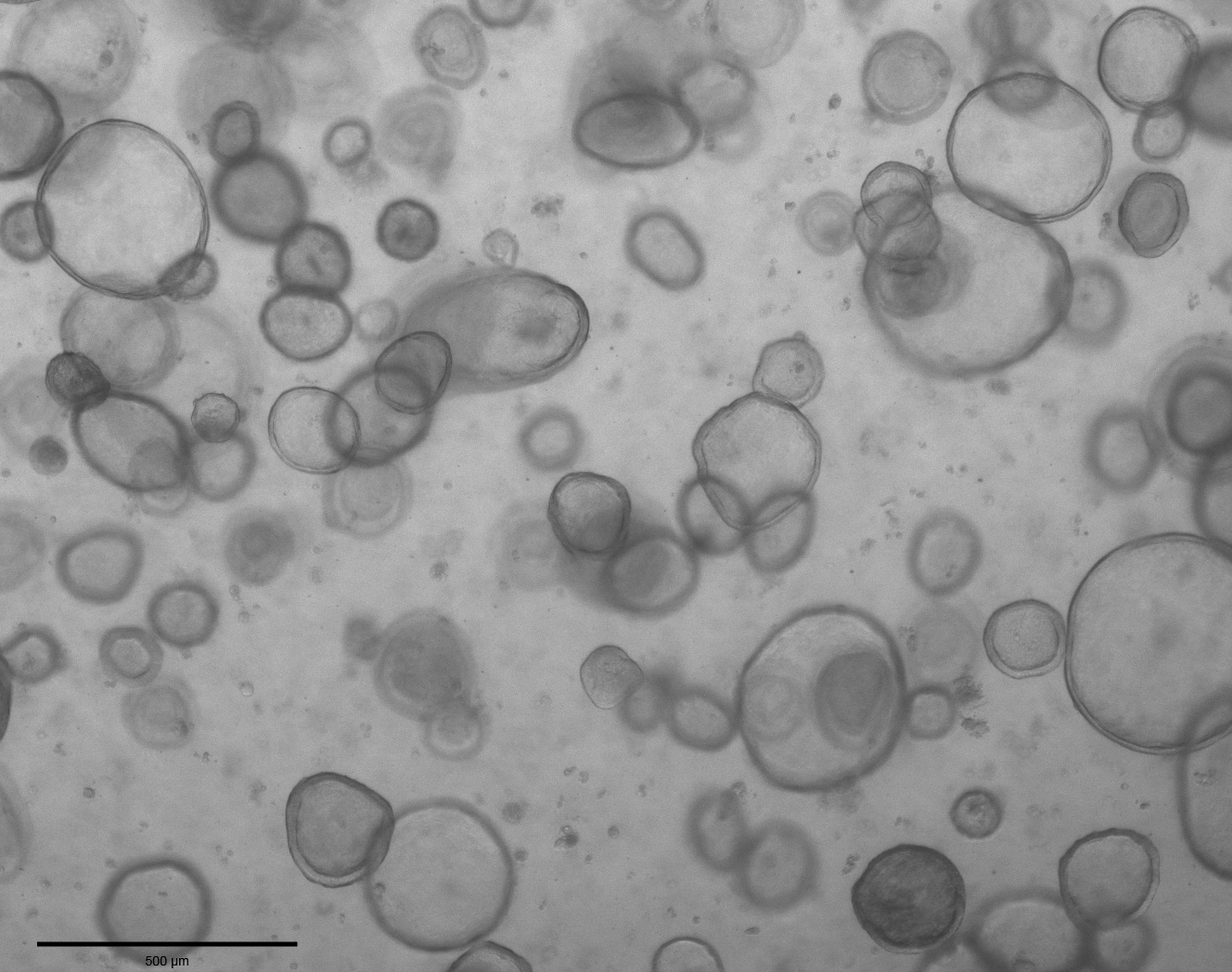

Supplement: Supplementary file 11 — Source data Fig. 6 [file 44318_2026_774_MOESM11_ESM.zip › Figure 6/C/untreated MP ALVO-EM.TIF]

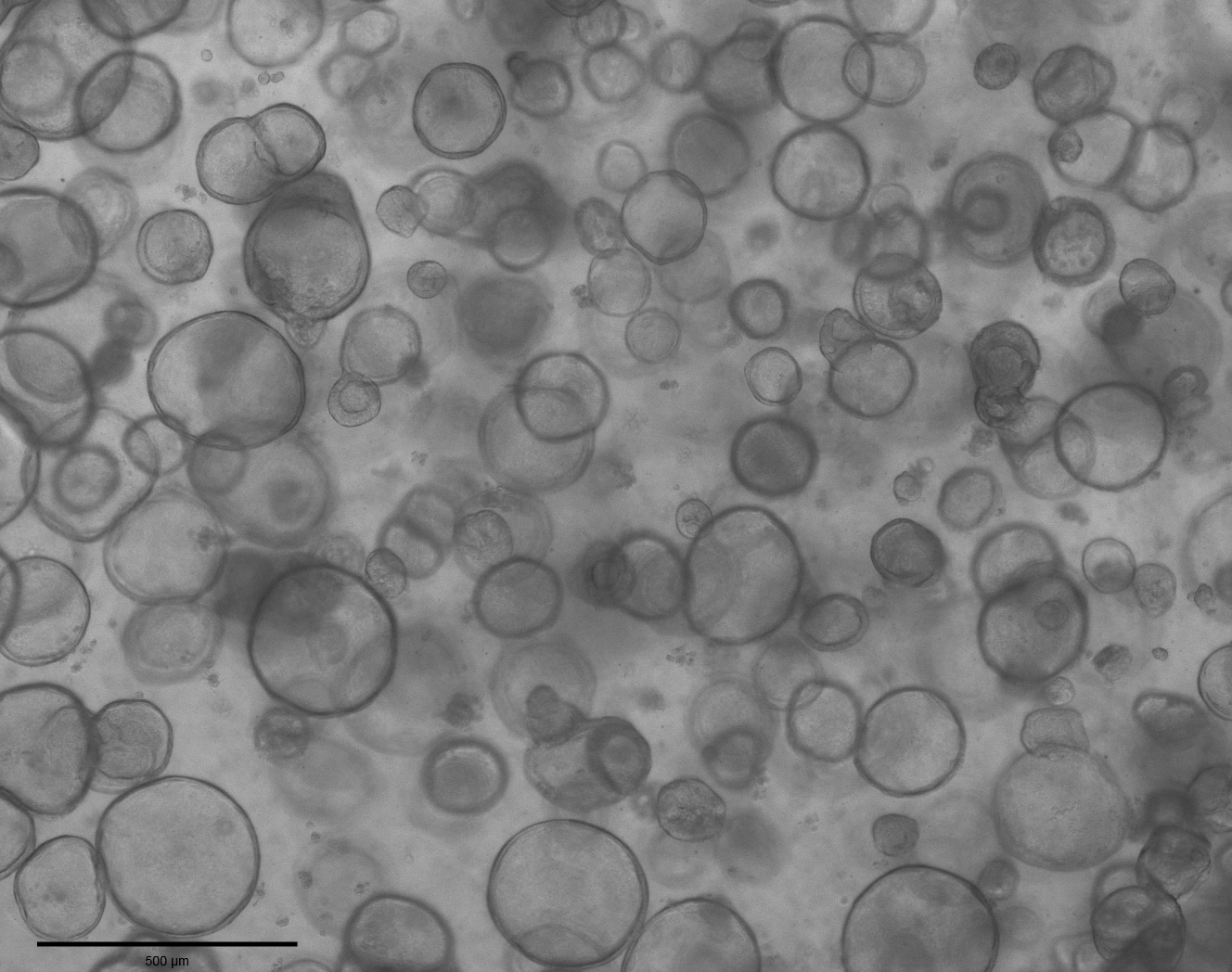

Supplement: Supplementary file 11 — Source data Fig. 6 [file 44318_2026_774_MOESM11_ESM.zip › Figure 6/C/treated MP ALVO-EM.TIF]
