# Supplementary material for: Interferon-γ selectively promotes survival of alveolar progenitor cells in a human lung organoid model
Source: EMBO J. 2026 Apr 16;45(10):3364–95. doi: 10.1038/s44318-026-00774-4 (PMC13187038; doi:10.1038/s44318-026-00774-4)
Supplement: Supplementary file 12 — Expanded View Figures [file 44318_2026_774_MOESM12_ESM.pdf]

Expanded View Figures

Figure EV1. Optimized ALVO conditions allow for long-term expansion of alveolar progenitor populations. Related to Fig. 1.

(**A**) IF images of AO organoids raised in AO media as a staining control, p0 organoids generated with our method, and p1 organoids after EPCAM+/NGFR- FACS sorting as described in Fig. 1A. Scale bar = 50 um. The right column shows a close-up of the area indicated by boxes in the left column. DAPI = nuclei; SFTPC = AT2 markers; KRT5 = basal cell marker; SCGB1A1 = club cell marker. (**B**) FACS plot showing sorting strategy of p0 d14 organoid-derived cells gated for DAPI-/EPCAM+ single cells. NGFR-/HT2-280+ (HT2+) and NGFR-/HT2-280- (HT2-) populations were sorted, plated in organoid cultures, and further analyzed in Fig. EV1C+E. (**C**) Brightfield images of HT2+ and HT2- sorted cells (see Fig. EV1B) cultured with HRG1 or EGF. (**D**) Dotplot showing gene expression of selected genes in different lung cell types. Expression data and cell type annotations were taken from the lung atlas Sikkema et al, 2023. (**E**) qPCR analysis of HT2+ and HT2- cells sorted from p0 organoids and cultured with HRG1 or EGF in p1 (see also EV1B+C). AOs are included for comparison. A two-sided paired *t*-test was performed on log-transformed raw values. Data were shown as mean ± SD. Exact *p* values are indicated. (**F**) FACS gating strategy for EPCAM+/NGFR- cell sorting of p0 organoids (left panel). Percentage of NGFR- cells within the EPCAM+ fraction (right panel). Data were represented as mean ± SD. (**G**) qPCR analysis of AT2 (*SFTPC*) and AT1 (*CAV1, AGER*) markers in ALVOs cultured in indicated media conditions for 14 days. A two-sided paired *t*-test was performed on log-transformed raw values. Data were shown as mean ± SD. Exact *p* values are indicated. (**H**) qPCR time course of AT2 (*SFTPA1* and *SFTPC*) and AT1 (*AGER*) markers in day 14-old ALVOs at indicated passages with whole lung lysate as reference (dotted line). (**I**) Dotplot of the top 15 differentially expressed genes (DEGs) for Seurat clusters 0, 1, and 2. Plot showing the clusters can be found in Fig. 1D.

 © The Author(s)

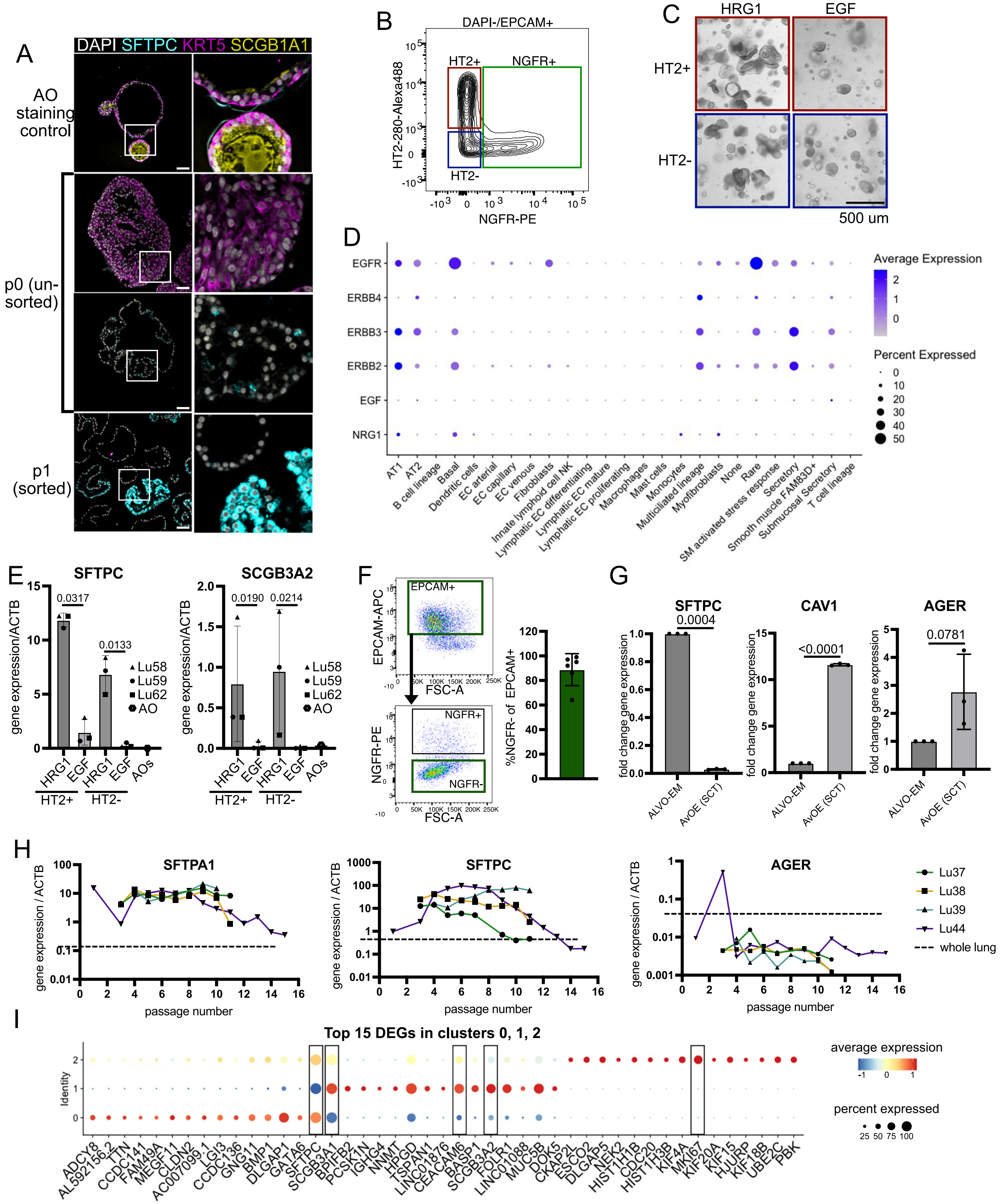
A
DAPI SFTPC KRT5 SCGB1A1
AO staining control
p0 (un-sorted)
p1 (sorted)
B
DAPI-/EPCAM+
HT2+
NGFR+
HT2-
HT2-280-Alexa488
NGFR-PE
C
HRG1
EGF
HT2+
HT2-
500 um
D
EGFR
ERBB4
ERBB3
ERBB2
EGF
NRG1
Average Expression
Percent Expressed
AT1
AT2
B cell lineage
Basal
Dendritic cells
EC arterial
EC capillary
EC venous
Fibroblasts
Innate lymphoid cell NK
Lymphatic EC differentiating
Lymphatic EC mature
Lymphatic EC proliferating
Macrophages
Mast cells
Monocytes
Multiciliated lineage
Myofibroblasts
None
Rare
SM activated stress response
Secretory
Smooth muscle FAM83D+
Submucosal Secretory
T cell lineage
E
SFTPC
SCGB3A2
gene expression/ACTB
0.0317
0.0133
0.0190
0.0214
Lu58
Lu59
Lu62
AO
HRG1
EGF
AOs
HT2+
HT2-
F
EPCAM+
EPCAM-APC
FSC-A
NGFR+
NGFR-
NGFR-PE
%NGFR- of EPCAM+
G
SFTPC
CAV1
AGER
0.0004
<0.0001
0.0781
fold change gene expression
ALVO-EM
AvOE (SCT)
H
SFTPA1
SFTPC
AGER
gene expression / ACTB
passage number
Lu37
Lu38
Lu39
Lu44
whole lung
I
Top 15 DEGs in clusters 0, 1, 2
Identity
average expression
percent expressed
ADCY8
AL592156.2
TTN
CCDC141
FAM49A
MEGF11
CLDN2
AC007099.1
LGI3
CCDC136
GNG11
BMP1
DLGAP1
GATA6
SFTPC
SCGB3A1
BPIFB2
PCSK1N
IGHG4
NNMT
HPGD
TSPAN1
LINC01876
CEACAM6
BASP1
SCGB3A2
FOLR1
LINC01088
MUC5B
DOK5
CKAP2L
ESCO2
DLGAP5
NEK2
HIST1H1B
CDC20
HIST1H3B
KIF4A
MKI67
KIF20A
KIF15
HJURP
KIF18B
UBE2C
PBK

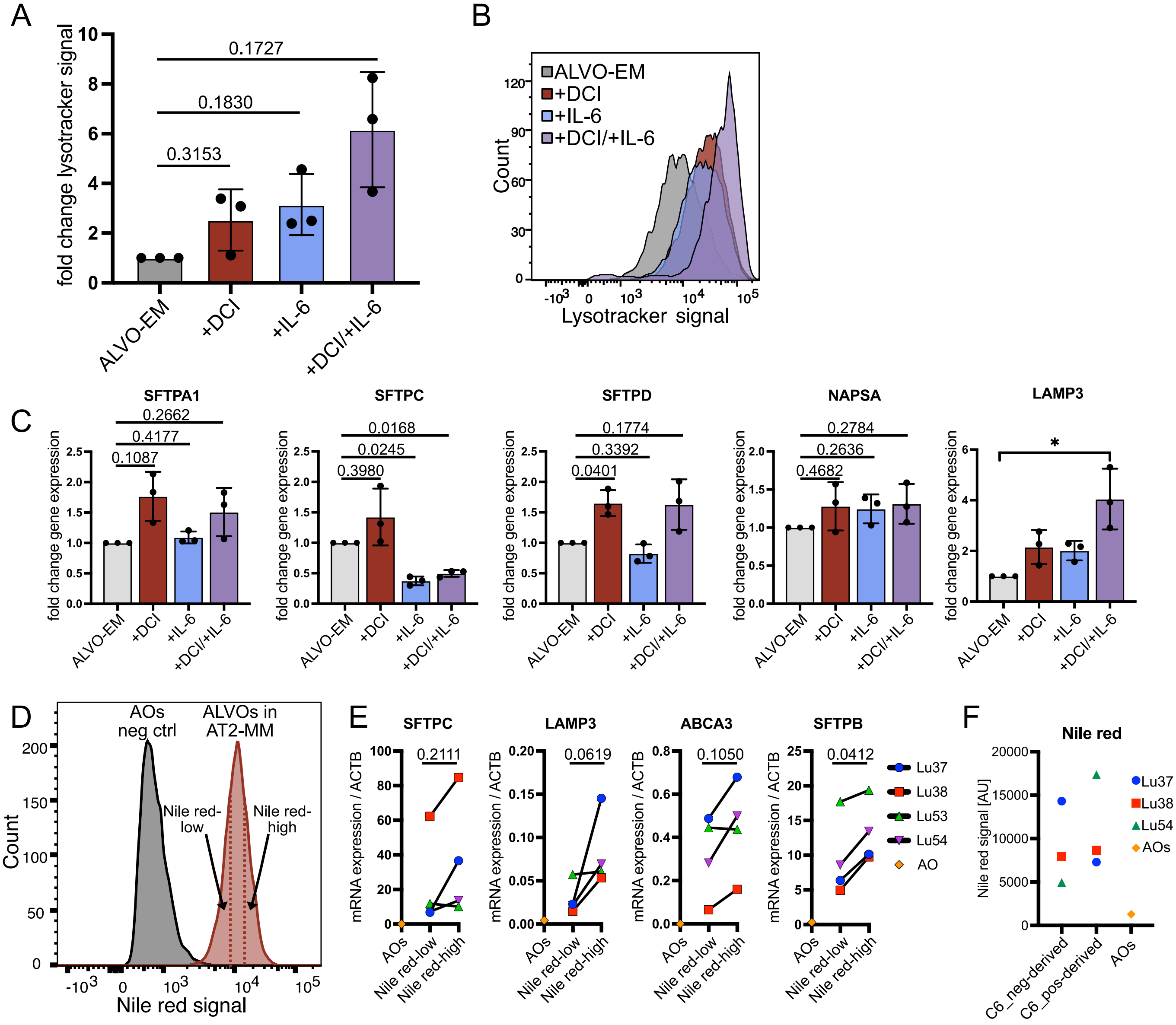

Figure EV2. Maturation media supports AT2 surfactant secretion with tubular myelin formation. Related to Fig. 2.

(**A**) Flow cytometry analysis of lysotracker signal in ALVOs cultured in the indicated conditions. Matched one-way ANOVA with Geisser–Greenhouse correction, followed by Dunnett's multiple comparisons versus control, was performed on log-transformed raw values. Data were shown as normalized mean ± SD. Exact adjusted *p* values are indicated. (**B**) Representative flow cytometry histogram showing Lyostracker signal distribution in indicated conditions. (**C**) qPCR analysis of AT2 markers in ALVOs cultured in the indicated conditions as outlined in Fig. 2A. Matched one-way ANOVA with Geisser–Greenhouse correction, followed by Dunnett's multiple comparisons versus control, was performed on log-transformed raw values. Data were shown as normalized mean ± SD. Exact adjusted *p* values are indicated. (**D**) Representative flow cytometry histogram showing Nile red signal distribution in AOs (negative control) and ALVOs cultured in AT2-MM. Nile red-low and -high cells are indicated by the dotted lines. (**E**) qPCR analysis of sorted Nile red-low and -high cells from ALVOs cultured in AT2-MM (also see panel **D**) and AOs (negative control). Two-sided paired *t*-tests were performed on log-transformed raw values. Exact *p* values are indicated. (**F**) Flow cytometry Nile red signal of C6- (neg) and C6+ (pos) derived cells matured in AT2-MM as outlined in Fig. 2A compared to AO-derived cells.

 © The Author(s)

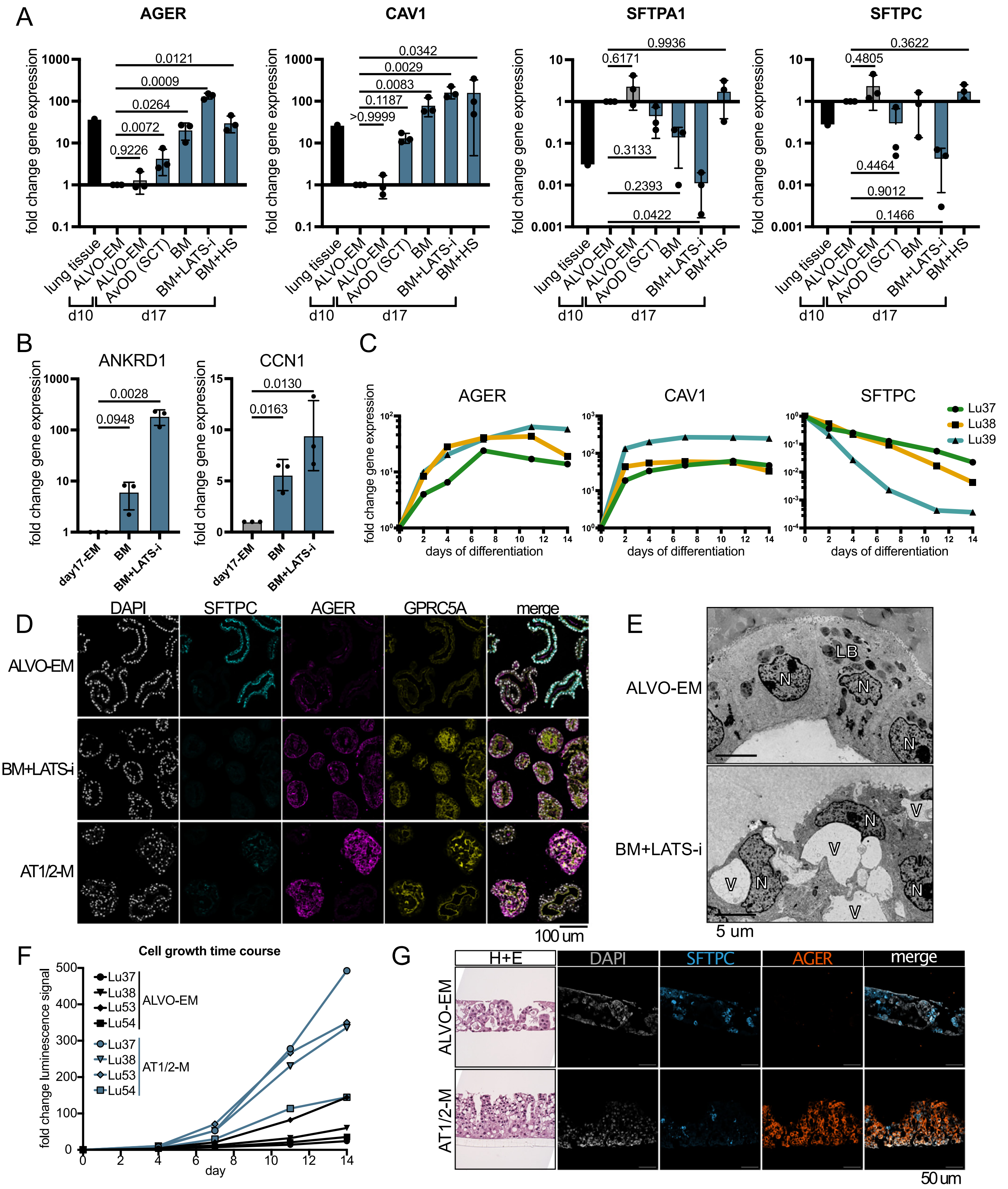
A
AGER
CAV1
SFTPA1
SFTPC
fold change gene expression
lung tissue
ALVO-EM
ALVO-EM
AvOD (SCT)
BM
BM+LATS-i
BM+HS
d10
d17
B
ANKRD1
CCN1
day17-EM
BM
BM+LATS-i
C
days of differentiation
Lu37
Lu38
Lu39
D
DAPI
SFTPC
AGER
GPRC5A
merge
ALVO-EM
BM+LATS-i
AT1/2-M
100 um
E
ALVO-EM
BM+LATS-i
LB
N
V
5 um
F
Cell growth time course
fold change luminescence signal
Lu37
Lu38
Lu53
Lu54
ALVO-EM
AT1/2-M
day
G
H+E
DAPI
SFTPC
AGER
merge
ALVO-EM
AT1/2-M
50 um

© The Author(s)

Figure EV3. Removal of proliferation factors and inhibition of LATS drives AT1 differentiation. Related to Fig. 3.

(**A**) qPCR analysis of AT1 (*AGER* and *CAV1*) and AT2 markers (*SFTPA1* and *SFTPC*) in ALVOs cultured in indicated conditions as outlined in Fig. 3A with whole lung tissue as reference. Matched one-way ANOVA with Geisser–Greenhouse correction followed by Dunnett's multiple comparisons versus control (ALVO-EM day 10) was performed on log-transformed raw values. Data were shown as normalized mean ± SD (normalized to ALVO-EM day 10). Exact adjusted *p* values are indicated. (**B**) qPCR analysis of YAP target genes in ALVOs cultured in the indicated conditions as outlined in Fig. 3A. Matched one-way ANOVA with Geisser–Greenhouse correction, followed by Dunnett's multiple comparisons versus control, was performed on log-transformed raw values. Data were shown as normalized mean ± SD. Exact adjusted *p* values are indicated. (**C**) qPCR time course of AT1 (*AGER* and *CAV1*) and AT2 markers (*SFTPC*) in the three indicated ALVO lines after switching to BM + LATS-i media following a 10-day expansion phase in ALVO-EM. (**D**) IF images of ALVOs cultured in the indicated conditions as outlined in Fig. 3A. DAPI = nuclei; SFTPC = AT2 marker; AGER and GPRC5A = AT1 marker. (**E**) Electron microscopy image of ALVOs cultured in ALVO-EM or BM + LATS-i as outlined in Fig. 3A. N nucleus, V vacuole, LB lamellar bodies. (**F**) Cell growth time course (cell titer glo) in four ALVO lines grown in ALVO-EM or AT1/2-M. Data were normalized to day 0. (**G**) Side-view brightfield (H + E staining) and IF images of cells in 2D ALI transwells cultured in the indicated media conditions. DAPI = nuclei; SFTPC = AT2 marker; AGER = AT1 marker.

 © The Author(s)

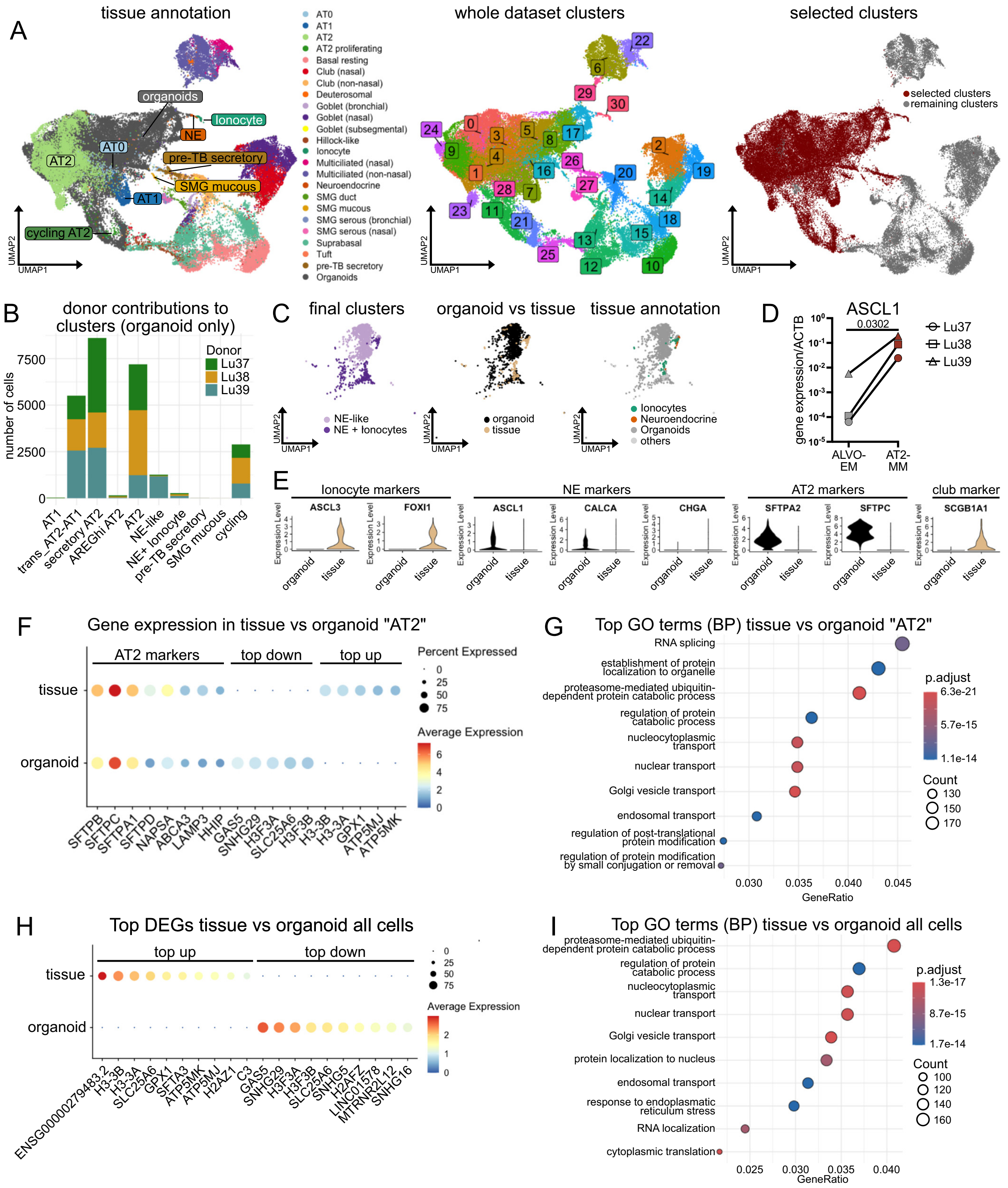
A
tissue annotation
whole dataset clusters
selected clusters
organoids
Ionocyte
NE
AT0
AT2
pre-TB secretory
SMG mucous
AT1
cycling AT2
AT0
AT1
AT2
AT2 proliferating
Basal resting
Club (nasal)
Club (non-nasal)
Deuterosomal
Goblet (bronchial)
Goblet (nasal)
Goblet (subsegmental)
Hillock-like
Ionocyte
Multiciliated (nasal)
Multiciliated (non-nasal)
Neuroendocrine
SMG duct
SMG mucous
SMG serous (bronchial)
SMG serous (nasal)
Suprabasal
Tuft
pre-TB secretory
Organoids
selected clusters
remaining clusters
UMAP1
UMAP2
B
donor contributions to clusters (organoid only)
number of cells
Donor
Lu37
Lu38
Lu39
AT1
trans_AT2-AT1
secretory AT2
AREGhi AT2
AT2
NE-like
NE+ Ionocyte
pre-TB secretory
SMG mucous
cycling
C
final clusters
organoid vs tissue
tissue annotation
NE-like
NE + Ionocytes
organoid
tissue
Ionocytes
Neuroendocrine
Organoids
others
D
ASCL1
0.0302
gene expression/ACTB
ALVO-EM
AT2-MM
Lu37
Lu38
Lu39
E
Ionocyte markers
NE markers
AT2 markers
club marker
ASCL3
FOXI1
ASCL1
CALCA
CHGA
SFTPA2
SFTPC
SCGB1A1
Expression Level
organoid
tissue
F
Gene expression in tissue vs organoid "AT2"
AT2 markers
top down
top up
tissue
organoid
Percent Expressed
Average Expression
SFTPB
SFTPC
SFTPA1
SFTPD
NAPSA
ABCA3
LAMP3
HHIP
GAS5
SNHG29
H3F3A
SLC25A6
H3F3B
H3-3B
H3-3A
GPX1
ATP5MJ
ATP5MK
G
Top GO terms (BP) tissue vs organoid "AT2"
RNA splicing
establishment of protein localization to organelle
proteasome-mediated ubiquitin-dependent protein catabolic process
regulation of protein catabolic process
nucleocytoplasmic transport
nuclear transport
Golgi vesicle transport
endosomal transport
regulation of post-translational protein modification
regulation of protein modification by small conjugation or removal
p.adjust
6.3e-21
5.7e-15
1.1e-14
Count
130
150
170
GeneRatio
H
Top DEGs tissue vs organoid all cells
top up
top down
tissue
organoid
Average Expression
ENSG00000279483.2
H3-3B
H3-3A
SLC25A6
GPX1
SFTA3
ATP5MK
ATP5MJ
H2AZ1
C3
GAS5
SNHG29
H3F3A
H3F3B
SLC25A6
SNHG5
H2AFZ
LINC01578
MTRNR2L12
SNHG16
I
Top GO terms (BP) tissue vs organoid all cells
proteasome-mediated ubiquitin-dependent protein catabolic process
regulation of protein catabolic process
nucleocytoplasmic transport
nuclear transport
Golgi vesicle transport
protein localization to nucleus
endosomal transport
response to endoplasmic reticulum stress
RNA localization
cytoplasmic translation
p.adjust
1.3e-17
8.7e-15
1.7e-14
Count
100
120
140
160
GeneRatio

Figure EV4. ScRNA-Seq comparison to lung tissue highlights regenerative phenotype of ALVOs. Related to Fig. 4.

(**A**) UMAPs showing integrated organoid/tissue data, colored by tissue annotations and organoids (left), seurat clusters (center), and selected clusters for subsetting (right). (**B**) Bar plot showing the contributions of each organoid line (donor) to the organoid fraction of each final cluster in the organoid/tissue subset. (**C**) UMAP subset of the "NE-like" and "NE + Ionocyte" clusters of the organoid/tissue subset, colored by final cluster (left), organoid vs tissue origin (center), and tissue annotation (right). (**D**) qPCR analysis of NE-fate transcription factor ASCL1 in ALVO-EM and AT1/2-M. A two-sided paired *t*-test was performed on log-transformed raw values. Exact *p* value is indicated. (**E**) Violin plots showing gene expression levels of selected genes in "NE-like" and "NE + Ionocyte" clusters. (**F**) Dotplot showing AT2 markers and top down- and up-regulated genes comparing organoid and tissue origin within the "AT2" cluster of the organoid/tissue subset. (**G**) Top ten GO terms (biological processes) of differentially expressed genes between tissue and organoid cells from the "AT2" cluster. (**H**) Dotplot showing top up- and down-regulated genes comparing organoid and tissue origin within the whole organoid/tissue subset. (**I**) Top ten GO terms (biological processes) of differentially expressed genes between tissue and organoid cells from the whole organoid/tissue subset.

 © The Author(s)

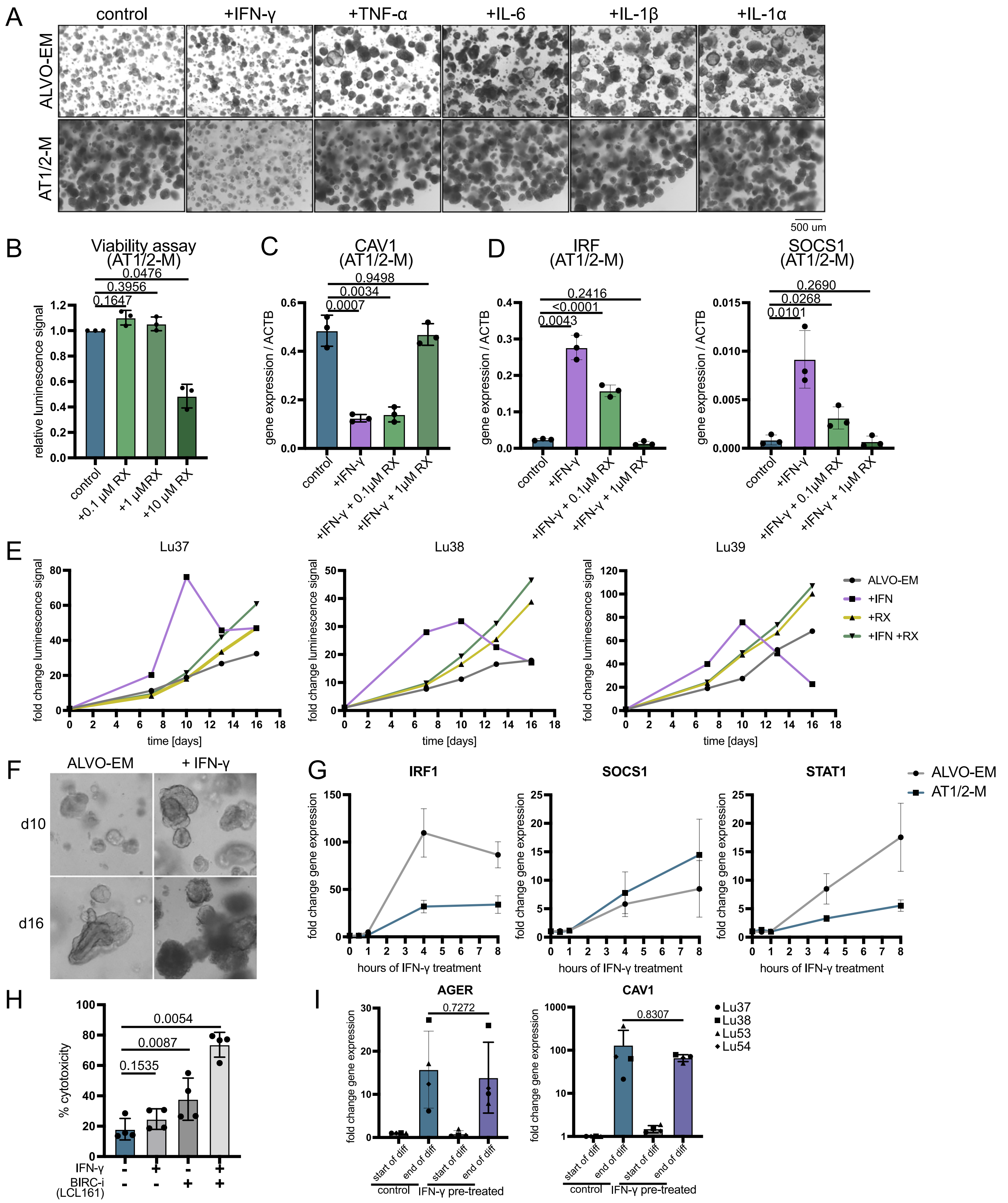
A
control
+IFN-γ
+TNF-α
+IL-6
+IL-1β
+IL-1α
ALVO-EM
AT1/2-M
500 um
B
Viability assay (AT1/2-M)
relative luminescence signal
0.1647
0.3956
0.0476
control
+0.1 μM RX
+1 μMRX
+10 μM RX
C
CAV1 (AT1/2-M)
gene expression / ACTB
0.0007
0.0034
0.9498
control
+IFN-γ
+IFN-γ + 0.1μM RX
+IFN-γ + 1μM RX
D
IRF (AT1/2-M)
0.0043
<0.0001
0.2416
SOCS1 (AT1/2-M)
0.0101
0.0268
0.2690
E
Lu37
Lu38
Lu39
fold change luminescence signal
time [days]
ALVO-EM
+IFN
+RX
+IFN +RX
F
ALVO-EM
+ IFN-γ
d10
d16
G
IRF1
SOCS1
STAT1
fold change gene expression
hours of IFN-γ treatment
ALVO-EM
AT1/2-M
H
% cytotoxicity
0.1535
0.0087
0.0054
IFN-γ
BIRC-i (LCL161)
I
AGER
0.7272
CAV1
0.8307
fold change gene expression
start of diff
end of diff
control
IFN-γ pre-treated
Lu37
Lu38
Lu53
Lu54

Figure EV5. IFN-γ selectively promotes alveolar progenitor growth while impairing AT1-like cells. Related to Fig. 5.

(**A**) Brightfield images of ALVOs grown in indicated media conditions in the absence (control) or presence of indicated cytokines for 14 days. (**B**) Viability assay of ALVOs cultured in AT1/2-M in the absence (control) and presence of indicated concentrations of RX for 14 days. Matched one-way ANOVA with Geisser–Greenhouse correction, followed by Dunnett's multiple comparisons versus control, was performed on log-transformed raw values. Data were shown as normalized mean ± SD. Exact adjusted *p* values are indicated. (**C**) qPCR analysis of *CAV1* in ALVOs cultured in AT1/2-M in the absence (control) or presence of IFN-γ (10 ng/ml) and RX (0.1 and 1 uM) for 14 days. Matched one-way ANOVA with Geisser–Greenhouse correction, followed by Dunnett's multiple comparisons versus control, was performed on log-transformed raw values. Data were shown as mean ± SD. Exact adjusted *p* values are indicated. (**D**) qPCR analysis of IFN-γ target genes in ALVOs cultured in AT1/2-M in the absence (control) or presence of IFN-γ (10 ng/ml) and RX (0.1 and 1 uM) for 14 days. Matched one-way ANOVA with Geisser–Greenhouse correction, followed by Dunnett's multiple comparisons versus control, was performed on log-transformed raw values. Data were shown as mean ± SD. Exact adjusted *p* values are indicated. (**E**) Viability assay time course of three ALVO lines cultured in ALVO-EM in the absence and presence of IFN-γ (10 ng/ml) and RX (1 uM). (**F**) Brightfield images of ALVOs cultured in ALVO-EM in the absence and presence of IFN-γ (10 ng/ml) at the indicated time points. (**G**) qPCR time course of IFN-γ target genes in ALVOs cultured in indicated media conditions in the presence of IFN-γ (10 ng/ml) normalized to their respective untreated controls. (**H**) Cytotoxicity assay of organoids in AT1/2-M with and without IFN-γ and the BIRC3 inhibitor LCL161 for 3 days. Matched one-way ANOVA with Geisser–Greenhouse correction, followed by Dunnett's multiple comparisons versus control, was performed on log-transformed raw values. Data were shown as normalized mean ± SD. Exact adjusted *p* values are indicated. (**I**) qPCR analysis of ALVOs that were not treated (control) or pretreated with IFN-γ from days 0–7, and differentiated in AT1/2-M subsequently from days 10–17. Two-sided paired *t*-tests were performed on log-transformed raw values between the indicated groups. Data were shown as normalized mean ± SD. Exact *p* values are indicated.

 © The Author(s)
